# Farnesyl-transferase inhibitors show synergistic anticancer effects in combination with novel *KRAS-G12C* inhibitors

**DOI:** 10.1038/s41416-024-02586-x

**Published:** 2024-01-26

**Authors:** Marcell Baranyi, Eszter Molnár, Luca Hegedűs, Zsófia Gábriel, Flóra Gréta Petényi, Fanni Bordás, Violetta Léner, Ivan Ranđelović, Mihály Cserepes, József Tóvári, Balázs Hegedűs, József Tímár

**Affiliations:** 1 Department of Pathology, Forensic and Insurance Medicine, Semmelweis University, H-1091 Budapest, Hungary; 2 KINETO Lab Ltd, H-1037 Budapest, Hungary; 3 Department of Thoracic Surgery, University Medicine Essen - Ruhrlandklinik, University Duisburg-Essen, D-45239 Essen, Germany; 4 Pázmány Péter Catholic University Faculty of Information Technology and Bionics, H-1083 Budapest, Hungary; 5 Department of Anatomy, Cell and Developmental Biology, Eötvös Loránd University, H-1117 Budapest, Hungary; 6 Department of Experimental Pharmacology and the National Tumor Biology Laboratory, National Institute of Oncology, H-1122 Budapest, Hungary

**Keywords:** Cancer therapy, Lung cancer, Gastrointestinal cancer

## Abstract

**Background:**

Inhibition of mutant *KRAS* challenged cancer research for decades. Recently, allele-specific inhibitors were approved for the treatment of *KRAS-G12C* mutant lung cancer. However, de novo and acquired resistance limit their efficacy and several combinations are in clinical development. Our study shows the potential of combining G12C inhibitors with farnesyl-transferase inhibitors.

**Methods:**

Combinations of clinically approved farnesyl-transferase inhibitors and KRAS G12C inhibitors are tested on human lung, colorectal and pancreatic adenocarcinoma cells in vitro in 2D, 3D and subcutaneous xenograft models of lung adenocarcinoma. Treatment effects on migration, proliferation, apoptosis, farnesylation and RAS signaling were measured by histopathological analyses, videomicroscopy, cell cycle analyses, immunoblot, immunofluorescence and RAS pulldown.

**Results:**

Combination of tipifarnib with sotorasib shows synergistic inhibitory effects on lung adenocarcinoma cells in vitro in 2D and 3D. Mechanistically, we present antiproliferative effect of the combination and interference with compensatory *HRAS* activation and *RHEB* and lamin farnesylation. Enhanced efficacy of sotorasib in combination with tipifarnib is recapitulated in the subcutaneous xenograft model of lung adenocarcinoma. Finally, combination of additional KRAS G1C and farnesyl-transferase inhibitors also shows synergism in lung, colorectal and pancreatic adenocarcinoma cellular models.

**Discussion:**

Our findings warrant the clinical exploration of *KRAS-G12C* inhibitors in combination with farnesyl-transferase inhibitors.

## Introduction

Mutations of *RAS* are estimated to be present in up to 19% of all malignancies, of which around 75% are *KRAS* mutations [1]. *KRAS* is one of the most commonly mutated driver oncogenes in lung, colorectal and pancreatic adenocarcinomas [2]. Though it was long considered “undruggable” following decades of unsuccessful targeting, this nickname was challenged by the recent development of *KRAS-G12C-specific* covalent inhibitors [3, 4]. Rapid optimization and further development led to the introduction of powerful drug candidates, of which sotorasib (AMG510) and adagrasib (MRTX849) have shown encouraging results in clinical trials [5, 6]. Sotorasib monotherapy resulted in a 37.1% objective response rate (ORR) in patients with *KRAS-G12C* mutant non-small cell lung cancer (NSCLC). The disease control rate (DCR) was 80.6% [7]. Besides, adagrasib monotherapy achieved similar results with 45% ORR and 96% DCR in NSCLC patients with *KRAS-G12C* mutations [6]. These results led to FDA approval of these drugs for lung adenocarcinoma [8, 9].

Though *KRAS-G12C* mutations are the most frequent in tumors of lung origin, it is also present in other solid tumors as well like colorectal or pancreatic adenocarcinomas. However, *KRAS-G12C* inhibitors administered as single agents showed far more modest activity in colorectal tumors: sotorasib only achieved 7.1% ORR with 73.8% DCR while adagrasib reached 17% ORR and 94% DCR [10, 11].

Clinical activity of sotorasib and adagrasib is unprecedented given that patients with *KRAS* mutant lung or colorectal adenocarcinomas had no or only limited access to targeted therapies as *KRAS* mutation is a negative predictive factor for all anti-*EGFR* therapies [12, 13]. However, the objective response rate is still relatively small, especially in patients with colorectal cancer. Furthermore, the first reports of clinically acquired resistance are already available [14]. Acquired resistance is a common event in response to targeted therapies like those targeting *BRAF*, *MEK*, and *EGFR* [15]. Observed resistance mechanisms show high variations from mutations altering the drug-binding pocket of *KRAS* protein through new oncogenic mutations (like *G12D*, *G13D*) affecting the *trans* alleles to copy number variations, activations or oncogenic fusions of *HER2*, *ALK*, *RET* or *BRAF* [14, 16]. Besides, feedback reactivation through wild-type alleles also seems to be one of the most prominent mechanisms [17].

A common strategy to improve efficacy and break down resistance is to apply combinational therapies. Indeed, currently several therapeutics are being tested in combination with adagrasib or sotorasib, like *SHP2* inhibitors, anti-*EGFR* therapeutics like cetuximab, afatinib or even immune checkpoint inhibitors and *CDK4* inhibitors [3, 18, 19]. First reports of adagrasib plus cetuximab and sotorasib plus panitumumab in colorectal cancer show encouraging results [20, 21]. However, new approaches are still urgently needed.

Historically, studies on *KRAS-driven* tumors – due to the perception of its target’s undruggability – initially focused on indirect approaches, like blocking downstream effectors (*RAF*, *MEK*), searching for synthetic lethal partners, or targeting *RAS* processing [3]. The latter was one of the earliest attempts that led to the often-cited failure in *KRAS* research: the introduction of farnesyl-transferase inhibitors. As *RAS* proteins are small GTPases that localize to the plasma membrane through lipid anchors (palmitoyl and farnesyl groups), inhibition of these posttranslational modifications seemed obvious. Preclinical experiments supported this approach, and soon selective and potent farnesyl-transferase inhibitors (FTi) entered the clinics [4, 22]. However, though their pharmacological and toxicity profiles were good and well tolerated, they failed to achieve clinically relevant antitumor effects in unselected patient populations [23]. Subsequent research found that *KRAS* and *NRAS* (but not *HRAS*) are subject to an alternative lipid modification, named geranylgeranylation, in case of farnesylation is blocked [24]. This type of adaptation hindered the application of farnesyl-transferase inhibitors for years. Recently, interest has been renewed towards FTis for the treatment of *HRAS* mutant solid tumors with encouraging results in phase II trials [25, 26], leading to FDA approval for *HRAS* mutant head and neck cancer, and for the Hutchinson-Gilford progeria syndrome, where the target is progerin [27].

Our group recently reviewed the rationale of using prenylation inhibitors (including FTis) for the treatment of *KRAS* mutant solid tumors [23]. Surprisingly, analyzing a publicly available database (https://depmap.org/portal/interactive/), we found that *KRAS* mutant lung adenocarcinomas (LUAD) were significantly more sensitive to FTis compared to *KRAS* wild-type cells [23]. Further investigation of this phenomenon based on *KRAS* mutational types reveals that cancer cell lines harboring *KRAS-G12C* mutations tend to be more prone to FTis effects. Notably, *KRAS-G12C* mutations take up to 40% of all *KRAS* mutant lung adenocarcinoma cases [2].

Based on these findings, we tested tipifarnib (that was recently granted Breakthrough Therapy Designation by the FDA) in combination with clinically applied *KRAS-G12C* inhibitors sotorasib and adagrasib on *KRAS-G12C* mutant LUAD cell lines, which show strong and robust synergistic antitumoral effects in 2D and 3D conditions. We also found synergistic effects using additional FTis and *KRAS-G12C* inhibitors as well as in *KRAS-G12C* mutant colorectal and pancreatic cancer models. Our in vitro findings are confirmed in in vivo xenograft experiments. Regarding the potential mechanism of action, we also show that FTis in combination with KRAS-G12C inhibitors disrupt compensatory HRAS reactivation, and block farnesylation of RHEB and nuclear lamina. This study provides a rationale for the exploration of a new combinational therapy for the treatment of *KRAS-G12C* mutant tumors, utilizing clinically approved FTis (tipifarnib, lonafarnib) with novel *KRAS-G12C* inhibitors.

## Results

### KRAS-G12C mutant LUAD cells are more sensitive towards FTis

**Fig. 1:**
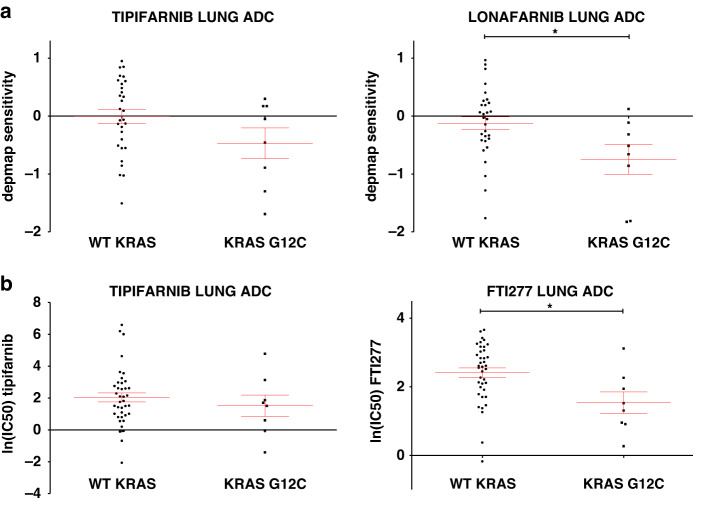
Sensitivity of lung adenocarcinoma cell lines to farnesyl-transferase inhibitors. **a** Data from PRISM Primary Repurposing Screen shows pooled and normalized (to the viability of all 572 or 562 cell lines tested in the screen) sensitivity values to tipifarnib and lonafarnib. Error bars represent SEM for 30 WT KRAS and 8 KRAS-G12C mutant cells. Statistical significance is marked by an asterisk and was determined by an unpaired *t*-test in the case of tipifarnib (*p* = 0.0896) and lonafarnib (*p* = 0.0182). **b** Data from the GDSC1 screen (Genomics of Drug Sensitivity in Cancer from cancerrxgene.org) shows the natural logarithm of IC50 to tipifarnib and FTI277. Error bars represent SEM for 39 WT KRAS and 8 KRAS-G12C mutant cells. Statistical significance is determined with *p* < 0.05 and is marked by an asterisk and was analyzed by unpaired *t*-test in the case of tipifarnib (*p* = 0.4594) results and Mann–Whitney *U* test in the case of FTI277 (*p* = 0.0143).

The Prism Repurposing Primary Screen (available from (https://depmap.org/portal/interactive/)) contains sensitivity values of hundreds of cell lines towards thousands of drugs applied at 2.5 µM in 5-days-long experiments [28]. Our previous in silico investigation using this screen indicated that lung adenocarcinoma cell lines harboring *KRAS* mutations are more sensitive to farnesyl-transferase inhibitors than those with wild-type *KRAS* [23]. In this study, we performed an exploratory investigation whether the *KRAS-G12C* mutation might be associated with different FTI sensitivities. We found a tendency for higher sensitivity of *KRAS-G12C* cells to farnesyl-transferase inhibitors compared to wild type (Fig. 1a).

In addition, when we investigated a distinct large-scale drug sensitivity database (GDSC1, Genomics of Drug Sensitivity in Cancer) available at (www.cancerrxgene.org) that utilizes IC50 values of the given drugs, we found that *KRAS-G12C* LUAD cell lines had significantly lower ln(IC50) values upon FTI277 treatment compared to wild type cells (Fig. 1b).

Interestingly, when we extended our analyses to other LUAD cells with other KRAS mutations, KRAS G12C mutant cells seemed to be the only mutational type that exhibited higher sensitivity towards FTis, although the small number of cells with non-G12C mutation limits the interpretation of this finding (Supplementary Fig. 1). Furthermore, when we compared FTi sensitivity based on the zygosity of KRAS mutations, there was an overall tendency for higher sensitivity of heterozygous KRAS mutant cell lines, however, differences did not reach statistical significance (Supplementary Fig. 1). Nevertheless, the low number of models with homozygous KRAS mutation decreases the statistical power in this exploratory analysis.

Based on these results, we decided to explore whether FTis can potentiate the two *KRAS-G12C-specific* drugs that are clinically approved and also one that is currently under clinical investigation [10, 11, 29].

### KRAS inhibitors with FTis are synergistic in LUAD in vitro

**Fig. 2:**
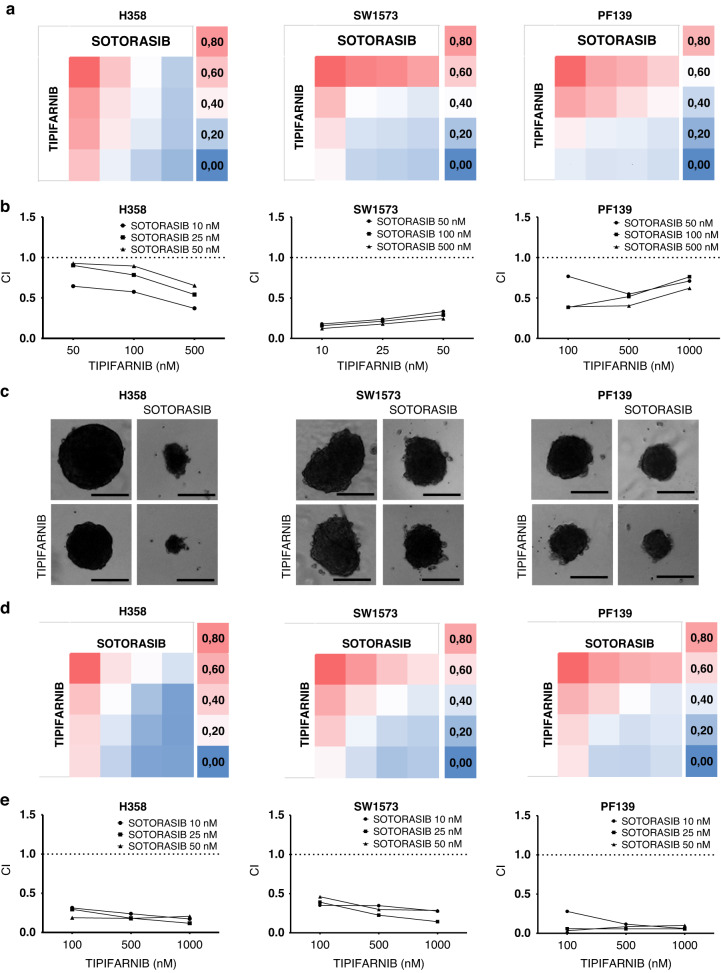
Combination of sotorasib and tipifarnib in three *KRAS-G12C* mutant lung adenocarcinoma cell lines. **a** Heatmaps of control-normalized cell viability values derived from 6-day-long 2D SRB tests. Treatment concentrations of each cell line can be seen in (**b**). Data is derived from three independent experiments. **b** Combinational index (CI) values of the 2D combinational experiments. CIs were calculated by CompuSyn Software. CI values less than 1 indicate synergy while values equal to or more than 1 represent additive or antagonistic effect, respectively. **c** Representative images of lung adenocarcinoma spheroids from 6-day-long 3D spheroid experiments exposed to 50 nM sotorasib and/or 100 nM tipifarnib treatment. Images were taken on the last day of the treatment. Scale bar means 0.2 mm. **d** Heatmaps of control-normalized spheroid volume data derived from 6-day-long 3D spheroid tests. The data shown is from three independent experiments. **e** Combinational index (CI) values of the 3D spheroid combinational experiments. Both 2D and 3D experiments resulted in robust synergistic combinational indexes.

For the determination of the combinational effects, we calculated drug interactions based on the Combinational Index (CI) theory [30]. This widely used method provides a quantitative definition of drug interactions (*CI* = *1* additive effect, *CI* > *1* antagonistic and *CI* < *1* synergistic effect). We first investigated sotorasib, which is already approved for clinical treatment of *KRAS-G12C* lung adenocarcinoma, in combination with tipifarnib, an FTi that was recently granted Breakthrough Therapy Designation for treatment of *HRAS* mutant head and neck squamous cell carcinoma [31]. To test combinational therapy, we first tested three *KRAS-G12C* mutant cell lines: H358 a sotorasib sensitive; SW1573, a sotorasib-resistant and a novel patient-derived cell line, PF139. 2D combinational SRB tests revealed robust synergistic interactions with sotorasib in all *KRAS-G12C* mutant LUAD cell lines. (Fig. 2a, b) We also validated our findings in multicellular tumor spheroids with the same drug combinations. 6-day-long treatment of tumor spheroids resulted in similarly strong synergistic drug interactions shown in Fig. 2c–e. Complete viability data showing means and standard error of mean (SEM) are in Supplementary Fig. 2. Besides cell viability and spheroid growth, we also demonstrated that the combination of sotorasib and tipifarnib significantly decreased the migratory activity of the PF139 cell line measuring haptotactic motility of single cells based on time-lapse videomicroscopy (Supplementary Fig. 3a). In H358 and SW1573 cells, only very limited single cell haptotic motility can be observed and thus no quantification is feasible. Accordingly, we used an alternative assay (wound healing) for investigation of invasion in all LUAD cells lines. Furthermore, we demonstrated that in case of PF139 and SW1573, combinational treatment slowed down cell migration in wound healing assay, while in case of H358 both sotorasib and combinational therapy blocked wound closure (Supplementary Fig. 3b).

### Combination of sotorasib and tipifarnib in vivo

Following in vitro experiments, we also investigated the effect of sotorasib and tipifarnib monotherapies or their combination on the growth of two human LUAD cell lines, H358 and SW1573, in subcutaneous xenograft models. We adjusted the in vivo dosage based on the differences observed in 2D conditions.

**Fig. 3:**
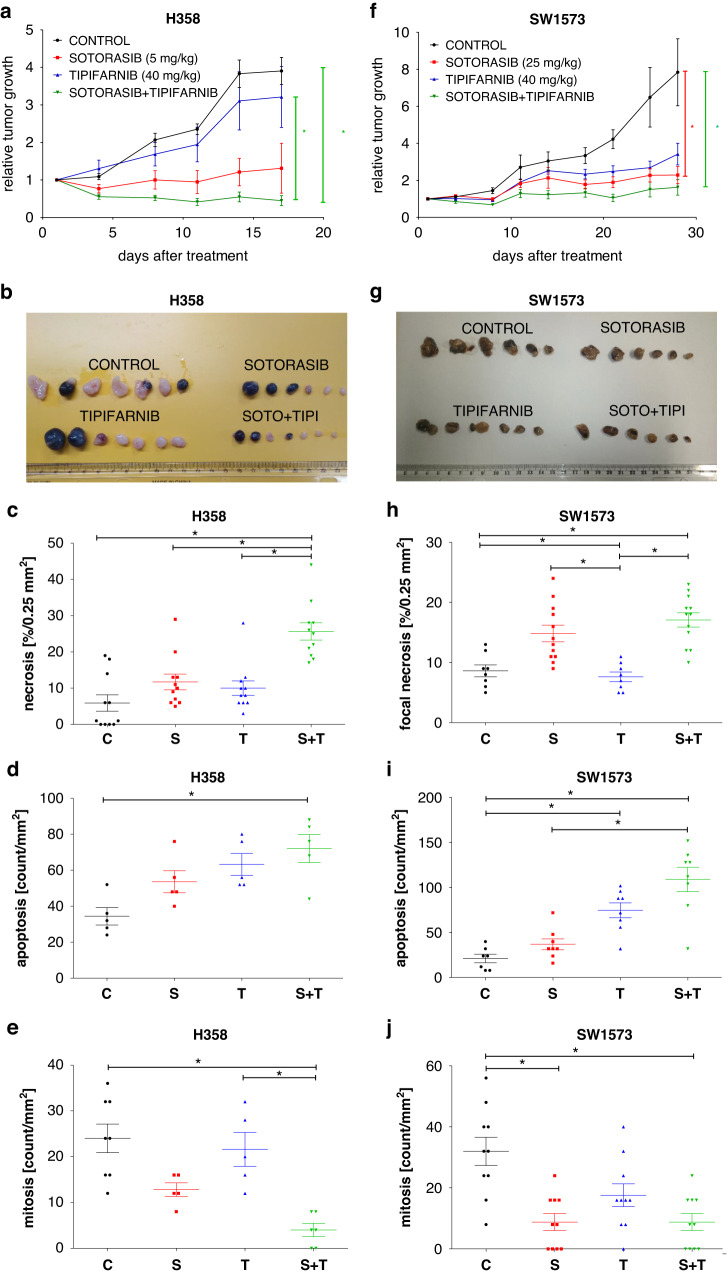
Combination of sotorasib and tipifarnib in the sotorasib-sensitive (H358) and sotorasib-resistant (SW1573) xenograft models of lung adenocarcinoma. H358 xenografts were given sotorasib (5 mg/kg i.p.) and/or tipifarnib (40 mg/kg i.p.), while SW1573 xenografts were treated with sotorasib (25 mg/kg i.p.) and/or tipifarnib (40 mg/kg i.p.) therapy. Treatment started after randomization when tumors reached approximately 100 mm^3^ (7 days following cell inoculation in the case of H358 xenografts and 26 days in the case of SW1573 tumors). Each treatment group contained 7 animals. The weekly treatment schedule was five days on and two days off. **a** Tumor volume was determined twice a week using a caliper. Relative tumor volume growth was normalized with each tumor’s starting volume on the day of the first treatment. Error bars represent SEM. In the case of H358 combinational treatment resulted in significantly smaller tumors compared to control and tipifarnib-treated tumors, while in SW1573 xenografts sotorasib monotherapy and combinational therapy resulted in significantly smaller tumors compared to control based on last day’s relative growth values. **b** Pictures of the harvested tumors. **c**–**e** Histopathological analysis of necrotic areas and frequency of apoptosis and mitosis. In H358 tumors, amount of necrotic areas in the combination treatment group were significantly higher compared to control and both monotherapies. A significant increase in apoptosis and decrease in mitosis can also be observed. In SW1573 tumors, focal necrotic areas were significantly larger in sotorasib and combination treatment group compared to control and tipifarnib group, while frequency of apoptotic cells was higher in tipifarnib and combination treatment group compared to control and sotorasib. Sotorasib and combination treatments also decreased frequency of mitotic cells compared to control. Asterisks marks statistically significant differences with *p* < 0.05. Statistical significance was tested with the Kruskal–Wallis test followed by Dunn’s multiple comparison test.

In the sotorasib-sensitive H358 model of LUAD, only the combinational treatment was able to exert statistically significant antitumor activities based on tumor measurements and histopathological analyses (Fig. 3, Supplementary Figs. 4–5). Specifically, combinational therapy significantly decreased tumor weights compared to control (measured following termination, shown in Supplementary Fig. 5a), and also tumor volumes (the last day’s relative growth values) compared to control and to tipifarnib monotherapy, but not to sotorasib monotherapy (Fig. 3a, b). However, histopathological evaluation (representative images in Supplementary Fig. 4) revealed that the percentage of the necrotic area of tumor tissues significantly increased upon combination treatment compared to both monotherapies and control (Fig. 3c) while the frequency of mitotic figures significantly decreased upon combination compared to tipifarnib monotherapy (Fig. 3e). Meanwhile, the frequency of apoptotic cells did not differ significantly compared to monotherapies (Fig. 3d).

In the sotorasib-resistant SW1573 model, sotorasib and combinational treatment exerted significant tumor growth inhibition compared to control xenografts based on the last day’s relative growth values (Fig. 3f, g). In the case of tumor weight measurement of SW1573 tumors, similarly to relative growth values, combinational treatment resulted in the lowest mean of tumor weights, though differences did not reach statistical significance (*p* = 0.122, shown in Supplementary Fig. 5a). However, the histopathological evaluation showed that the percentage of focal necrotic areas in tumor tissues significantly increased upon combinational treatment compared to tipifarnib monotherapy (Fig. 3h) while the frequency of apoptotic cells induced by combinational therapy increased significantly compared to sotorasib treatment. (Fig. 3i) Meanwhile, there was no change in mitotic cell frequency in combinational treatment compared to monotherapies. (Fig. 3j)

Animal body weight losses never reached 10% during the study and were around a maximum of 5% loss (Supplementary Fig. 5b). Histopathological analysis of the liver and kidney tissues did not reveal morphologic alterations either in the animals receiving monotherapies or in the animals receiving combinations, even at the higher dose of sotorasib administration. Furthermore, the fine structure of the kidney as assessed by PAS basement membrane staining similarly did not show light microscopically detectable alterations (Supplementary Fig. 5c–e). These results suggest that the combination of sotorasib and tipifarnib is well tolerated in experimental animals.

### Sotorasib and tipifarnib interferes with RAS signaling

**Fig. 4:**
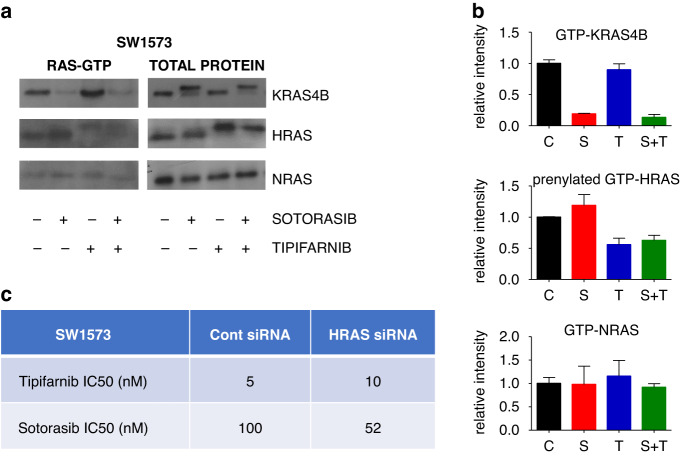
*RAS* protein levels and activation following 48-hour-long treatment with sotorasib (100 nM) and/or tipifarnib (500 nM). **a** Images show representative blots of the *RAS* proteins. *RAS*-GTP stands for only GTP-bound, active *RAS* proteins while “total protein” shows blots from the whole cell lysates. **b** Graphs represent normalized levels of GTP-bound *KRAS4B*, *HRAS* and *NRAS*, respectively. Note that the graph shows only changes of the prenylated fraction of *HRAS*. Protein levels were normalized to control. **c** Changes of IC50 values upon transfection with non-targeting and *HRAS*-targeting siRNA after 6-day-long treatment with tipifarnib and sotorasib. All data are from three independent experiments. Graphs show mean with error bars representing SEM.

We investigated the protein farnesylation and *RAS* activation in the G12C-inhibitor-resistant SW1573 cell line since only mutated *KRAS* gene is expressed in it (homozygous), and therefore all changes are pertinent to the *KRAS* mutant oncoprotein. Western blot analyses revealed that tipifarnib treatment did not interfere with *KRAS4B* and *NRAS*, while effectively blocked farnesylation of *HRAS* proteins both in single agent and combination therapy, where a small, but clear shift in electrophoretic mobility could be observed (upper band represents unfarnesylated proteins) (Fig. 4a).

In line with these results, *GTP-KRAS4B* level only decreased upon sotorasib and combination treatment. The level of *GTP-NRAS* was low and did not change upon treatment. Interestingly, the level of *GTP-HRAS* increased upon sotorasib treatment likely compensating for the decrease of active *KRAS4B*. Furthermore, *GTP-HRAS* upon tipifarnib and combinational treatment was mainly found at the non-prenylated fraction thus not able to contribute properly to *RAS-mediated* signaling [23]. Collectively, the combination reduced both active *GTP-KRAS4B* and *GTP-HRAS* protein levels in SW1573 cells. In addition, the same pattern of changes in *RAS* activation was observed upon investigation of the MIAPACA2 pancreatic cell line, shown in Supplementary Fig. 6.

To further investigate the role of *HRAS* in the mechanism of action of the synergism in combination therapy, we decreased *HRAS* levels by siRNA and determined changes in IC50 values of sotorasib and tipifarnib in SW1573 cells (Fig. 4c). We found that upon siRNA knockdown of *HRAS*, IC50 of sotorasib decreased from 100 to 52 nM, while IC50 of tipifarnib increased from 5 to 10 nM. Validation of successful *HRAS* knockdown is shown in Supplementary Fig. 7. Our in vitro finding is also supported by the in silico investigation of the CRISPR knockout data from (https://depmap.org/portal/interactive/) indicating an inverse correlation between *KRAS* and *HRAS* dependency in *KRAS-G12C* mutant lung adenocarcinoma cell lines (Supplementary Figure 8).

Marked changes in *KRAS4B* and *HRAS* activation led us to test drug effects on the downstream effectors of *RAS* following 48-hour-long treatment. (Supplementary Fig. 9) Interestingly, we found substantial, although cell-line dependent changes in the activation of the major *RAS*-related *RAF/MEK/ERK* and *PI3K/AKT/mTOR* pathways. The level of phosphorylated *ERK1/2* and phosphorylated *S6* decreased in the majority of the cell lines upon combination treatment, while monotherapies resulted in diverse responses. (Supplementary Fig. 9) Of note, we found that farnesylation of *RHEB*, a key protein in the *PI3K/AKT/mTOR* signaling network, was also strongly inhibited by both tipifarnib and the combination treatments. (Supplementary Fig. 9) Additionally, we investigated the impact of treatments on another important oncogenic pathway, the SRC/FAK signaling. We found a modest increase in autophosphorylation of FAK protein upon sotorasib and combinational treatments in SW1573 and PF139 cells. In the meantime, all treatments reduced autophosphorylation of FAK in H358 cell line. Notably, no treatment induced changes in SRC activation in any of the three cell models (Supplementary Fig. 10).

### Tipifarnib or combination block cytokinesis and PTM of lamins

Next, we investigated changes in cell cycle distribution and markers of apoptosis and proliferation. Importantly, cell line specific patterns were observed among the three LUAD cell lines. In the H358 cell line, sotorasib and combinational treatment drastically increased the ratio of the cells in the subG1 and strongly reduced it in the G2/M and S phases. (Supplementary Fig. 11a)

**Fig. 5:**
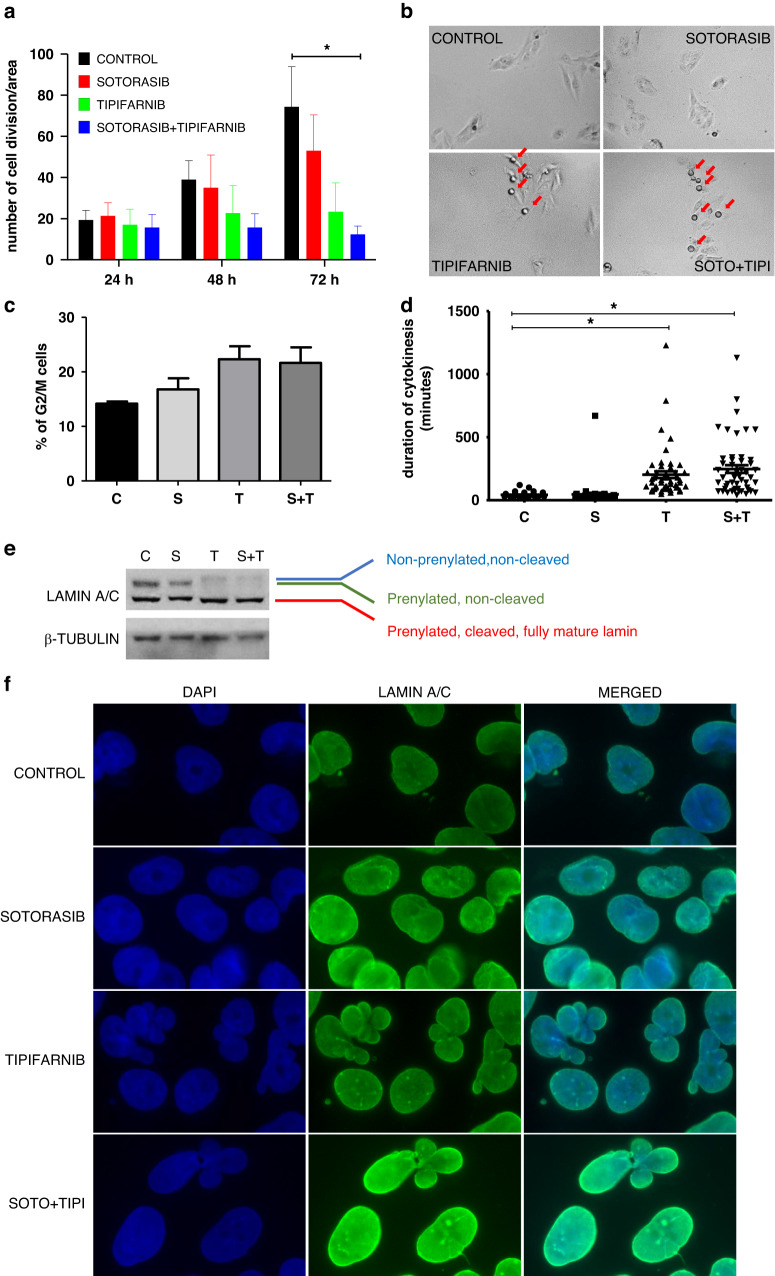
Investigation of the proliferative activity of SW1573 cell line upon treatment and alterations in the laminar network. **a** Number of cell divisions during a 72-hour-long treatment with sotorasib (100 nM) and/or tipifarnib (500 nM). Combination treatment induced a significant decrease in cell divisions on the last day of the treatment (*N* = 3, total number of cell divisions from three independent experiments from three fields of view/experiment. Asterisks marks statistically significant differences with *p* < 0.05. Statistical significance was tested with the Kruskal-Wallis test followed by Dunn’s multiple comparison test.). **b** Representative pictures from the videomicroscopic analysis show accumulation of cells unable to execute cytokinesis after treatment with tipifarnib alone or in combination with sotorasib. **c** Changes in M phase following treatment using a modified, M-phase preserving protocol of cell cycle investigation. **d** Duration of cytokinesis was measured following 48-hour-long treatment. Both tipifarnib and the combination treatment significantly increased the duration of cytokinesis. **e** Western blot analyses of Lamin A/C changes. Tipifarnib and the combination treatment resulted in the appearance of an upper third band (representative of the non-prenylated, non-cleaved lamins) while the level of prenylated, non-cleaved lamins diminished. **f** Immunofluorescence labeling of Lamin A/C proteins. DAPI (blue) staining reveals lobular cell nuclear morphology upon tipifarnib and combinational treatment. These treatments also lead to the accumulation of distinct spots of Lamin A/C (green) in the nucleus. All data shown derives from or is representative of three independent experiments. Graphs show mean with error bars representing SEM.

Tipifarnib and combinational treatment induced apoptosis in SW1573 and H358 cell lines and also slightly elevated the ratio of cells in the G2/M phase. We also confirmed these findings by Western blot detection of cleaved *PARP*. (Supplementary Fig. 11) *PCNA* (marker of proliferation) levels decreased upon sotorasib, and more effectively by combinational treatment in H358 and PF139 cell lines. However, in the resistant SW1573 cell line *PCNA* was present in a higher amount and none of the treatments could effectively lower its expression. (Supplementary Fig. 11b, c), so we decided to further analyze other potential molecular mechanisms in this cell line. Accordingly, we performed time-lapse video microscopy of the 72-hour-long treatment with sotorasib and/or tipifarnib. Manual tracking of cytokinesis revealed that all types of treatment inhibited mitotic activity of SW1573 cells but only combination treatment resulted in statistically significant changes. (Fig. 5a) Additionally, after 24 h of treatment, the round-up cells preparing for cytokinesis remained for a longer interval in this stage and demonstrated a significant delay in cytokinesis in cells exposed to tipifarnib or the combination treatment. (Fig. 5b, d)

Furthermore, performing a modified, M phase preserving protocol of cell cycle experiment, a pronounced though not significant (*p* = 0.0602) increase in the ratio of G2/M phase cells was revealed upon tipifarnib or combination treatment. (Fig. 5c) As *lamin A/C* proteins, the main components of the nuclear laminar network, are also farnesylated we investigated whether changes in this structure might contribute to the delayed cytokinesis. Western blot analyses confirmed successful inhibition of *lamin* farnesylation, with a clear loss of farnesylated, non-cleaved *lamin* proteins, though the level of prenylated and fully mature, cleaved *lamin* apparently did not show any changes. (Fig. 5e) However, immunofluorescent labeling of *lamin A/C* showed strong changes in its morphology upon tipifarnib and combination treatments. First, an accumulation of the nuclei with a lobular phenotype could be observed (Fig. 5f). Second, distinct spots of *lamin A/C* appeared in the nuclei of cells exposed to tipifarnib or combination treatment indicating an intranuclear accumulation of *lamin* proteins (Fig. 5f).

### Various combinations of KRAS inhibitors with FTis are synergistic in LUAD, CRC and PDAC

**Fig. 6:**
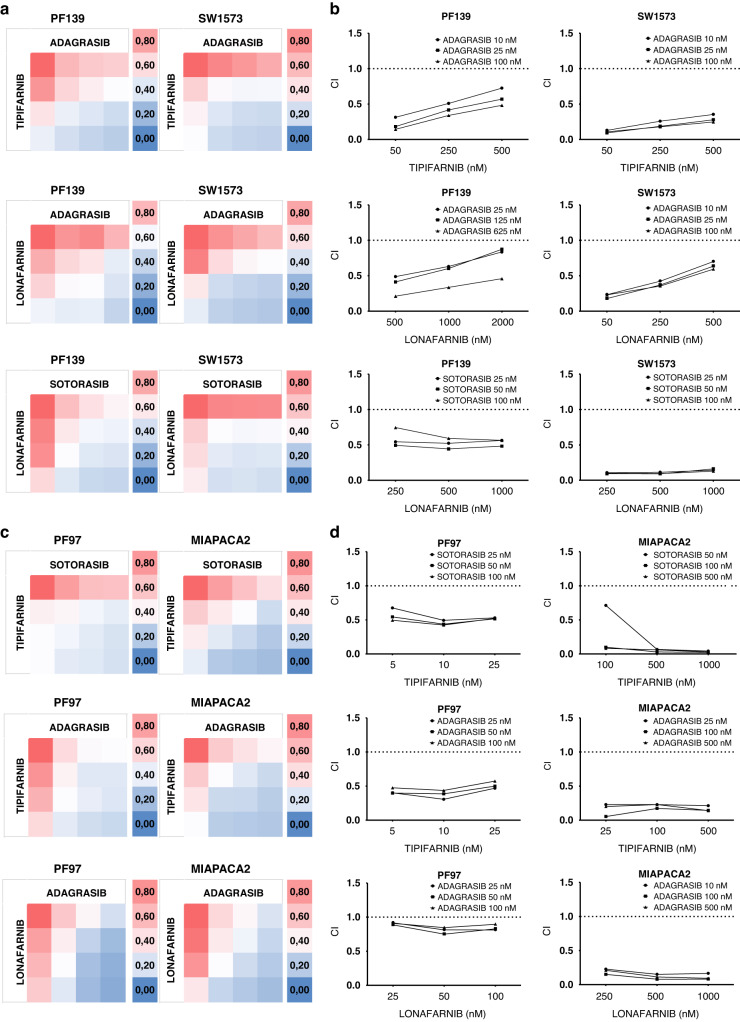
Combination treatments with various combinations of KRAS-G12C inhibitors and FTis in KRAS-G12C mutant lung, colorectal and pancreatic adenocarcinoma cell lines. **a**, **c** Heatmaps of control-normalized cell viability values derived from 6-day-long 2D SRB tests. PF139 and SW1573 are lung, while PF97 colorectal and MIAPACA2 pancreatic adenocarcinoma cell lines. Treatment concentrations used for each cell line are shown in (**b**, **d**). Data is derived from three independent experiments. **b**, **d** Combinational index (CI) values of the 2D combinational experiments. CIs were calculated by CompuSyn Software. CI values less than 1 indicate synergy while values equal to or more than 1 represent additive or antagonistic effect, respectively.

We tested additional combinational settings on SW1573 and PF139 LUAD cell lines using the *KRAS-G12C* inhibitor adagrasib and the farnesyl-transferase inhibitor lonafarnib, which also showed synergistic drug effects. (Fig. 6a, b) Complete viability data showing means and standard error of mean (SEM) are in Supplementary Fig. 12a.

Additionally, even though *KRAS-G12C* mutations are the most frequent in lung adenocarcinoma, there is a small percentage of tumors with this specific mutation in other tumor types as well. Accordingly, we tested the effect of sotorasib and adagrasib in combination with both farnesyl-transferase inhibitors (tipifarnib, lonafarnib) on the *KRAS-G12C* mutant pancreatic cancer cell line MIAPACA2 and a novel patient-derived colorectal cancer cell line PF97. We found that every combinational set-up had a synergistic effect in both cell lines (Fig. 6). Complete viability data showing means and SEM are in Supplementary Fig. 12a.

## Discussion

Our study proposes a novel combination therapy for *KRAS-G12C* mutant cancers utilizing two clinically approved drug classes, namely *KRAS-G12C* inhibitors and farnesyl-transferase inhibitors. These combinations showed synergistic antitumoral effects in all five *KRAS-G12C* mutant cancer models in 2D in vitro experiments and the findings were recapitulated in lung adenocarcinoma 3D models in vitro and in xenografts in vivo.

The landscape of the treatment of *KRAS-G12C* mutant solid tumors has changed with the introduction of covalent, allele-specific *KRAS-G12C* inhibitors, among which sotorasib and adagrasib have already been clinically approved [9]. As there was no targeted therapy for *KRAS* mutant tumors before, the clinical activity of sotorasib and adagrasib is considered as a breakthrough in these tumor types. However, objective response rate is still relatively small (30–45% in lung and 7–20% in colorectal adenocarcinoma) [6, 7, 10, 11]. Thus, numerous clinical trials are underway investigating potential drug combinations that can improve the antitumoral efficacy of these novel *KRAS-G12C* inhibitors [18, 32]. These attempts include vertical or horizontal combinational approaches and also combinations of KRAS inhibitors with immunomodulatory therapies [32]. Vertical combinations rely on the observation that blockage of mutant KRAS signaling leads to compensatory reactivation of the pathway [17, 33]. Simultaneous inhibition of upstream elements like blockage of EGFR can diminish this effect and lead to synergistic antitumoral effects [34, 35]. Another promising approach is targeting SHP2 in combination with KRAS G12C inhibitors [32, 35]. Notably, SHP2 has been proven to be an important upstream regulator for proper KRAS signalization and is a promising candidate for combinational therapy, as its blockade in parallel with KRAS inhibitors can inhibit wild-type RAS signaling [35]. Although they act at a different level, SOS1 inhibitors exhibit similar effects when used in combination with KRAS targeting [32].

Horizontal combinations include the targeting of PI3K/AKT/mTOR signaling, which is a well-established escape route upon RAS-MEK-ERK inhibition [32, 36, 37]. Furthermore, preclinical evidence revealed synergistic antitumoral effects of cell cycle inhibitors (e.g. cyclin-dependent kinase inhibitors) combined with KRAS G12C inhibitors [32, 38].

In addition, mutant KRAS signaling has a wide range of immunomodulatory effects [32]. In line with these observations, preclinical evidence shows that the application of KRAS inhibition along with immunomodulatory therapies like immune-checkpoint inhibitors shows enhanced antitumoral effects [32, 39, 40].

However, none of these preclinical investigations or clinical trials has employed farnesyl-transferase inhibitors, likely due to the long history of the failures of FTis as monotherapies in *KRAS* mutant cancers [3, 32].

Our analysis of publicly available databases showed that lung adenocarcinoma cell lines harboring *KRAS-G12C* mutation tend to be more sensitive to FTis than *KRAS* wild-type cells.

Interestingly, comparison of different types of KRAS mutations revealed that KRAS G12C shows the highest sensitivity towards FTis, however, small numbers of LUAD cell lines with other mutations limit the interpretation of this finding. Regarding zigosity, there was a tendency for higher sensitivity towards FTis of heterozygous KRAS mutant cell lines when compared to cells possessing only the mutant allele, however, the number of homozygous cell models is rather small. Of note, upon blockage of farnesylation, KRAS and NRAS (but not HRAS) proteins undergo an alternative prenylation, named geranyl-geranylation and are therefore not affected by FTis [23, 24]. However, FTi sensitivity may also be influenced by the differential rewiring of RAS signalization and vulnerabilities. For instance, we identified a significant inverse correlation between KRAS and HRAS dependency on KRAS G12 mutant LUAD cell lines. Furthermore, it has been shown that intrinsic GTPase activity of KRAS G12C protein is retained in contrast with most of the other mutant KRAS proteins [32, 41], which may lead to distinct outcomes. Dependency on HRAS signaling, RHEB activity in PI3K/AKT signaling and at least 63 farnesylated proteins may modulate sensitivity towards FTis. Our findings warrant a more detailed examination of modulatory factors of sensitivity towards farnesyl-transferase inhibition.

Our observations on FTi sensitivity led us to combine tipifarnib, a clinically approved potent farnesyl-transferase inhibitor with sotorasib, a novel *KRAS-G12C* inhibitor in lung adenocarcinoma cell lines. Surprisingly, strong synergistic antitumor drug interactions were demonstrated in all cell lines investigated in adherent conditions. This finding was confirmed in 3D spheroid models. We identified similar drug interactions with additional relevant drugs in clinical development, namely adagrasib and lonafarnib. Besides the antiproliferative effect, the combination of sotorasib and tipifarnib also exerted antimigratory activity in vitro in single cell motility of PF139 cells as well as in wound healing assays. In the case of H358, sotorasib and combinational treatment drastically blocked wound closure, likely due to the combined antimigratory and antiproliferative effects. In H358 cells, tipifarnib alone did not reduce wound closure, in line with previous observation that the impact of tipifarnib on cell migration is cell-type dependent [42].

Furthermore, though both sotorasib and tipifarnib monotherapy achieved tumor growth inhibition in our xenograft experiments, the combination exerted the most pronounced impact on tumor growth in two distinct xenograft models of human lung adenocarcinoma utilizing the sotorasib-sensitive H358 and sotorasib-resistant SW1573 cells. Regarding tipifarnib dosage, we utilized double the amount of the corresponding human dose (600 or 900 mg b.i.d, equivalent to ~17 or 25 mg/kg daily in a 70 kg human), adjusted to the higher growth rate of xenograft tumors. Notably, this dosage is still half of the dose that could be found in previous studies [43]. In the case of sotorasib dosage, the human dose is 960 mg daily [7], which is equivalent to 14 mg/kg per day in a 70 kg human. When adjusting dosage for our xenograft models, we also took into consideration different sensitivities of the utilized cell lines towards KRAS inhibition. Notably, H358 is a commonly utilized model for *KRAS-G12C* inhibition, as it exhibits particularly high sensitivity towards *KRAS-G12C* inhibitors both in vitro and in vivo, which shows growth inhibition even upon 3 mg/kg dosage [39, 44]. In contrast, SW1573 showed high level of resistance towards *KRAS* inhibition in vitro in 2D conditions and was also found to be refractory in a study utilizing adagrasib [38, 44]. In line with these results, growth of H358 xenografts was limited upon as small as 5 mg/kg i.p. treatment, while 25 mg/kg dose was necessary to achieve a similar effect in SW1573 tumors. Interestingly, we showed that tipifarnib monotherapy is also able to exert a pronounced inhibitory effect on SW1573 xenografts, which is – to our knowledge – the first demonstration of FTi anticancer effects in vivo on KRAS G12C mutant model. Importantly, combined treatments were able to reduce the volume of H358 xenografts and suppress the growth of SW1573 tumors. Furthermore, even though neither tumor volumes measured with caliper nor tumor weights showed significant differences between monotherapies and combinational therapy, histopathological analyses revealed enhanced efficacy of the latter. Specifically, in the case of H358 xenografts, tumors in the combinational treatment group exhibited a significantly higher percentage of necrotic area compared to tumors in both single agent groups. This finding leads to the conclusion that a lower amount of living tumor tissue can be found in combinational therapy group, which could not be differentiated when measuring tumor volume or weight. In addition, the density of mitotic cells was also significantly lower in tumors treated with combinational therapy compared to sotorasib-treated tumors. Histology of SW1573 tumor samples revealed that the combination induced a significantly higher amount of focal necrosis compared to tipifarnib-treated tumors and increased frequency of apoptotic cells significantly higher compared to sotorasib-treated tumors. In this model, no difference was found in mitotic cell density between single agent and combination treatments. In summary, based on histopathological analyses, the combined application of KRAS G12C and farnesyl-transferase inhibition showed significant anti-tumoral effects in both models not only compared to the control but also compared to monotherapies.

However, such combinations may have unique toxicities. In our mice xenograft model, we have not seen major weight loss upon combination therapies (5–10%) and we have not detected histopathological alterations in the liver and kidney tissues, two organs that are affected by FTIs and G12C inhibitors in monotherapy clinical trials [9, 25, 27, 45]. One has to consider that the treatment of animals was less than a month, while the treatments last for several months in the clinical setting. Accordingly, chronic toxicity studies are necessary to test possible side effects in experimental animals, before entering the clinics. Also, one limitation of this present work is the lack of pharmacodynamic and pharmacokinetics data for the treatments, however, they are available for the monotherapies in early clinical studies [9, 46].

To investigate the potential cellular mechanism of this synergism, we performed cell cycle analysis and studied the expression of apoptosis and proliferation markers. We observed that the G2/M phase was slightly elevated in all cell lines upon FTi treatment in line with previous observations [47, 48]. Interestingly, using a modified, M phase preserving protocol, a more pronounced elevation of the G2/M phase was observed in SW1573 cell line suggesting that a significant amount of cells get stuck in the mitotic phase upon farnesyl-transferase inhibition. The ratio of the cells in the SubG1 phase also increased in H358 and SW1573 cell lines upon both FTi and combinational treatments and cleavage of the *PARP* protein was also detected in these samples. Investigation of the putative proliferation marker *PCNA* showed that both monotherapies and the combination effectively blocked proliferation in H358 and PF139 cell lines. Thus, treatment with FTis and *KRAS-G12C* inhibitors can exert antiproliferative, proapoptotic and even anti-migratory effects in vitro.

The analysis of the downstream signaling network of *KRAS* revealed diverse, cell line-dependent effects of the single agent and combination treatments on the *PI3K/AKT/mTOR* and RAF/*MEK*/*ERK* cascades. In line with previous results, we also observed a marked reduction of *ERK* phosphorylation upon *KRAS-G12C* inhibition [35, 38, 39]. However, in contrast with other’s findings, we showed that *S6* activation did not decrease upon *KRAS-G12C* blockade in two out of three of our LUAD cellular models. Only combinational treatment was able to reduce *S6* phosphorylation in all cell lines, which is an important hallmark of response to drugs [49]. Moreover, we also demonstrated successful inhibition of *RHEB* farnesylation, a key regulatory protein in the *PI3K/AKT/mTOR* pathway. The importance of this finding is underlined by the fact that horizontal combinational inhibition of *RAS* pathways is also proposed as a potential therapeutic approach [32]. Interestingly, investigation of activation of FAK-SRC signalization revealed increased FAK autophosphorylation upon sotorasib and combination treatments in PF139 and SW1573 cell lines, while all treatments reduced p-FAK in H358. Notably, it was previously shown that oncogenic RAS can lead to dephosphorylation of FAK at the 397 tyrosine through PIN1 and PTP-PEST leading to increased migration and enhanced wound closure [50]. Blockage of oncogenic KRAS can thereby relieve this negative signal, possibly leading to decreased migratory capacity.

Our investigation on the prenylation of the three classical *RAS* proteins revealed that FTi treatment successfully inhibited prenylation of *HRAS* but did not affect *KRAS* and *NRAS*, in line with previous observations [24]. Specifically investigating the activation of *RAS* proteins by assessing the level of GTP-bound *RAS*, we demonstrated the decrease by *KRAS-G12C* inhibition in parallel with compensatory *HRAS* activation. *GTP-NRAS* was changed by none of the treatments. Notably, *GTP-HRAS* was mainly found in the non-functional unprenylated form upon FTi treatment [23]. Our finding confirms previous demonstration of compensatory activation of wild-type *RAS* proteins upon *KRAS-G12C* targeting [35]. In a study by Ryan and colleagues, six cell lines showed elevated *GTP-NRAS* and *GTP-HRAS* upon KRAS G12C inhibitor ARS1620 treatment [35]. In our study, we also found elevated *GTP-HRAS* level but no change was detected in the amount of *GTP-NRAS* either in SW1573 or in MIAPACA2 cells. These experiments indicated that while in sotorasib-treated cells *HRAS* and in tipifarnib-treated cells *KRAS* can contribute to *RAS-mediated* signaling, the combination blocks the activity of both proteins and in turn leads to the observed synergism. This finding is particularly important as it was shown that inhibition of oncogenic *KRAS* can result in the reactivation of *RAS*-controlled pathways through wild-type *RAS* proteins [33]. However, it should be noted that both models used for the investigation of the role of HRAS in targeting the reactivation of the RAS pathway were homozygous for KRAS G12C mutations. Thus, further studies are needed to explore this phenomenon in a heterozygous setting where wild type KRAS proteins are also present. Furthermore, we also targeted *HRAS* with siRNA and managed to sensitize cells towards *KRAS-G12C* inhibition with a concomitant decrease in sensitivity against farnesyl-transferase inhibition. This data further supports the role of *HRAS* in the observed synergistic drug interactions. In addition, our in silico analysis found a significant inverse correlation between *HRAS* and *KRAS* dependency in *KRAS-G12C* mutant lung adenocarcinoma suggesting that these two proteins can play a compensatory role in tumor growth.

Investigation of the sotorasib-resistant SW1573 cell line with videomicroscopy revealed strong inhibition of cell division upon *KRAS-G12C* inhibition and FTi treatment. Notably, the combination resulted in the most pronounced, significant decrease in the number of successful cell divisions suggesting that synergistic drug interactions work through inhibition of proliferation. Furthermore, the videomicroscopic analysis revealed that many cells exhibited a dramatic delay in cytokinesis upon FTi and combinational treatment. These cells that struggled to perform cytokinesis likely contributed to the increase in the ratio of G2/M phase cells in cell cycle experiments. Several farnesylated proteins are important in regulating the mechanical aspects of cell division including centromere-associated proteins (*CENP-E, CENP-F*) and the laminar network of the nucleus [51, 52]. Indeed, we demonstrated that inhibition of farnesylation of *lamin A/C* proteins by tipifarnib initiated intranuclear accumulation of *lamin* and aberrant nuclear morphology, similar to the effects of another FTi, lonafarnib [53].

Since current G12C inhibitors are less active in colorectal or pancreatic cancers [19, 54], we also tested the combination of FTis and *KRAS-G12C* inhibitors on pancreatic and colorectal adenocarcinoma cells. Similar to our observations on lung adenocarcinoma, we found that all drug combinations resulted in strong synergistic antitumoral drug interaction in both types of human cancer cell lines in vitro.

It should be noted that due to pleiotropic nature of FTi action (as showed in the list of farnesylated proteins), a detailed investigation of each FTi target protein (e.g. through siRNA knockdown) in the synergistic drug interaction is not feasible. However, we identified several factors that can contribute to the observed synergism. These particular molecular mechanisms are also targets of other proposed combinational therapies that are curently in clinical development.

One synergistic interaction is the parallel targeting of PI3K/mTOR pathway by inhibiting the farnesylation of RHEB. The blockage of RHEB can induce changes similar to the mTOR inhibitor everolimus, which is currently evaluated in combination with sotorasib for KRAS G12C mutant tumors in clinical trials [32]. Indeed, we observed enhanced inhibition of S6 activation (downstream of RHEB and mTOR) upon the combinational therapy compared to monotherapies in all cell lines investigated, indicating that this combination is successful in reducing the activity of the PI3K/mTOR pathway.

Second, we have shown that FTi can reduce the compensatory reactivation of wild-type RAS proteins, namely HRAS, which is a known target of farnesyl-transferase inhibitors. Targeting the reactivation is also subject to clinical trials, specifically the combination of KRAS G12C inhibitors with SHP2 (e.g TNO155 with both sotorasib and adagrasib or RMC-4360 with sotorasib) and SOS1 inhibitors (e.g. BI1701963 with adagrasib), upstream elements in the RAS pathway [32]. The blockage of oncogenic KRAS G12C proteins relieves negative feedback loops on proteins upstream of RAS proteins leading to increased flux of the signaling through wild-type RAS proteins [17, 33]. Targeting of wild-type RAS activation by specific inhibition of proteins involved in RAS activation like SOS1 and SHP2 was shown to result in robust and profound synergistic combinational drug interactions when applied in parallel with KRAS inhibitors [32]. We also observed compensatory reactivation of HRAS (but not NRAS) using RAS-pulldown assays. We also demonstrated that HRAS farnesylation was blocked in two cell lines with distinct tissue of origin (SW1573 LUAD and MIAPACA2 PDAC cell lines). Furthermore, silencing HRAS with siRNA in the SW1573 cell line led to an increase of the IC50 of tipifarnib in parallel with a reduction of the IC50 of sotorasib. In addition, further supporting the role of HRAS, we found an inverse correlation between HRAS and KRAS dependency in KRAS mutant cell lines based on a publicly available database containing CRISPR sensitivity data (https://depmap.org/portal/interactive/).

Third, we provided evidence that farnesyl-transferase inhibition delayed cell cycle progression likely at least in part due to its impact on nuclear lamina. The combination of cell cycle inhibitors and KRAS G12C inhibition is indeed in clinical development using the cyclin D inhibitor palbociclib [32]. Of note, a couple of centromere-associated proteins (CENPE, CENPF) are also subject to farnesylation and may contribute to the synergistic effect.

In summary, the utilization of FTis in KRAS combinational therapies shares common mechanisms with three combinational approaches that are currently being evaluated in clinical trials.

The limitations of the current study are that our cancer models do not recapitulate intratumoral heterogeneity, like patient-derived organoids or patient-derived xenograft tumors. Intertumoral heterogeneity was addressed by means of utilizing five different stable cell lines from three different tissue of origins, of which two are novel patient-derived cell lines established by our group. Furthermore, the subcutaneous xenograft model – although its evaluation is more straightforward than more sophisticated orthotopic or transgenic models – cannot recapitulate important features as immune response or orthotopic cellular environment. Nevertheless, we performed in vivo experiments using two distinct cellular models – one heterozygous KRAS G12C inhibition-sensitive and one homozygous KRAS-G12C-inhibition resistant model. Another limitation of this manuscript is that we could not provide proof-of-concept for one circumscribed mechanism of action for the observed synergistic drug interactions. Furthermore, we could not precisely decipher the mechanism of action of the proposed combination due to the pleiotropic nature of farnesyl-transferase inhibition. Nevertheless, we demonstrated that several potentially contributing farnesylated proteins are inhibited in the proposed treatment including the FTi-sensitive wild-type HRAS and RHEB proteins or lamins.

Besides its limitations, our work clearly demonstrates that the application of FTis can potentiate antitumoral effects of novel KRAS G12C inhibitors and suggests that combination of farnesyl-transferase and *KRAS-G12C* inhibitors should be explored in the clinical setting both in *KRAS-G12C* mutant lung adenocarcinoma and in other *KRAS-G12C* mutant tumors.

## Methods

### In silico analysis of drug sensitivity from public databases

First, we analyzed drug sensitivity data from a publicly available website (https://depmap.org/portal/interactive/) mirroring information from the Prism Repurposing Primary Screen [28]. This database contains experimental data with various inhibitors at 2.5 µM concentration upon 5 days of treatment. Sensitivity data were grouped and downloaded according to *KRAS* mutational status for two different farnesyl-transferase inhibitors, lonafarnib and tipifarnib.

Next, IC50 of FTis from the GDSC1 dataset (tipifarnib and FTI277 were available for this drug class) was downloaded from (www.cancerrxgene.org) for all lung adenocarcinoma cell lines available. IC50 data was determined following 72-hour-long treatment with various concentrations of the given drug. Data was transformed using the *x=ln(IC50)* formula and was grouped based on *KRAS* mutational status. The type of *KRAS* mutation and zygosity was determined for each cell line based on the Cellosaurus database (https://cellosaurus.org).

Finally, *HRAS* and *KRAS* CRISPR knockout sensitivity data was obtained from https://depmap.org. Filters were set for NSCLC adenocarcinoma cell lines and combined CRISPR sensitivity values (DepMap 22Q2 Public+Score, Chronos) were downloaded.

A list of farnesylated proteins was obtained from (www.uniprot.org) and provided in Supplementary Table 2. Briefly, filters were set for human genes and lipidation for post-transcriptional modifications and then screened for S-farnesyl cysteine-modified entries.

### Cells and reagents

Three *KRAS-G12C* mutant lung adenocarcinoma, one colorectal and one pancreatic cancer cell line were involved in this study shown in Supplementary Table 1. H358 and SW1573 were purchased from ATCC. PF97 and PF139 cells were established from malignant pleural effusion samples in cooperation with the West German Biobank Essen as described earlier [16]. The patients provided written informed consent and the experiments were approved by the Ethics Committee of the University Hospital Essen (#18-8208-BO).

Lonafarnib and tipifarnib (for in vitro experiments) were purchased from Sigma (St. Louis, MO, USA), while sotorasib, adagrasib and tipifarnib (for in vivo experiments) were obtained from Medchemexpress. For in vitro experiments, drugs were dissolved in dimethyl sulfoxide (DMSO) in 10 mM stock concentration and stored at −80 °C.

### Cell viability assays

Cells at 70-80% confluence were trypsinized and counted using Luna II Automated Cell Counter (Logos Biosystems). Cells were plated in 5000-10000 cells/well density (depending on the growth rate of the given cell line) on a 24-well plate. The medium was replaced by fresh medium supplemented with inhibitors the next day. After 6 days, wells were washed with DPBS (Dulbecco’s phosphate-buffered saline) (Lonza) and the cells were fixed with 10% trichloroacetic acid and stained with Sulphorodamine-B (SRB) (Sigma) dye for 15 min. Plates were repeatedly washed with 1% acetic acid to remove excess dye. Protein-bound SRB was dissolved in 10 mM Tris buffer (pH = 7.4) and OD (optical density) was measured at 570 nm using a microplate reader (EL800, BioTec Instruments, Winooski, VT, USA). OD values were normalized to control. Transformed data were used to calculate combinational index (CI) [30]. The data shown are the results of three independent experiments. Heatmaps were prepared using Microsoft Excel software.

### 3D spheroid assays

Cells were seeded at 3000 cells/well density at the inner 60 polyHEMA-coated wells in 200 µl medium, while the outer wells were filled with DPBS (Lonza) to avoid evaporation. Cells were concentrated at the bottom of the well enhancing single spheroid formation by centrifugation at 540 RCF (relative centrifugal field). Within 24 h the cells aggregated into spheroids and were treated by the addition of a further 100 µl medium supplemented with inhibitors. Each concentration group contained three spheroids. Each spheroid was photographed using a ToupCam XW 3MP microscope camera on the first and sixth days of the treatment. Images were analyzed using ImageJ software with a modified script from [55]. Briefly, the script measures the area of the 2D projections of the spheroids. Results were manually reviewed, and area values were used to calculate the radius and volume of spheroids using the formula *V* = 4/3 × π × radius^3^. The data shown are the results of three independent experiments.

### In vivo experiments

H358 and SW1573 human lung adenocarcinoma cells (5 × 10^6^ and 1 × 10^6^, respectively) were subcutaneously injected in 28-28 female SCID (Severe combined immunodeficiency) female mice. Cells were injected in 200 µl DMEM:Matrigel (Corning, Corning, NY, USA) mixture (ratio 1:1) based on preliminary experiments. When tumors reached approximately 100 mm^3^ (H358 7 days, SW1573 26 days after injection), animals were randomized using minimization method (7 animals/group) and treated intraperitoneally daily except for weekends. Group size was determined by previous experiments. Drugs were dissolved in 60% DPBS, 34% PEG300 (polyethylene-glycol), 5% DMSO and 1% Tween80. H358 xenografts were treated with 5 mg/kg AMG510; and 40 mg/kg tipifarnib, while SW1573 xenografts were treated with 25 mg/kg AMG510 and 40 mg/kg tipifarnib. Controls received vehicle. The subcutaneous tumors were measured with a caliper and tumor volumes were calculated with the formula *V* = (length × widths^2^) × π/6 and expressed in mm^3^. Death of animals or damage of the subcutaneous tumors were set as exclusion criteria. The H358 experiment was terminated after 18 days and SW1573 after 25 days of treatment. Tumors were measured upon harvest and were fixed in 4% paraformaldehyde overnight, embedded into paraffin along with liver and kidney. Using hematoxylin and eosin stained slides tumor tissues were evaluated for necrotic area proportion, mitotic figure and apoptotic cell densities using calibrated 10/10 ocular grid morphometry performed by an experienced pathologist (JTímár). For evaluation of kidney tissue integrity sections have been labelled also by PAS for basement membrane and protein content (Supplementary Fig. 5).

56 female SCID mice were obtained from KINETO Lab’s animal house (Budapest, Korányi str. 1, H-1121, Hungary, license number: PEI-001-1715-2/2015, 24/07/2015). All experiments were carried out following the Guidelines for Animal Experiments. Permission Number: PE/EA/401-7/2020, 23/04/2020. The animal experiment was performed according to the regulations and recommendations from directive 2010/63/EU of the European Parliament and the Council of the European Union on the protection of animals used for scientific purposes. The health status of the mice was assessed by the animal house staff of KINETO Lab.

### Active RAS pulldown experiments

GTP-bound *RAS* samples were prepared using Active Ras Pull-Down and Detection Kit (Cat.no: 16117) from Thermo Fisher Scientific according to the manufacturer’s protocol. Further details are described in Supplementary Materials and Methods.

### Videomicroscopy

Videomicroscopy was performed using SW1573 cells at 5000 for cell division analyses and PF139 cells at 8000 cells/well density in 24-well plates for motility experiments. The day after seeding, the medium was replaced with fresh medium containing tipifarnib (500 nM), sotorasib (100 nM) or both. Three independent wells were included for each treatment group. One field of view was photographed in each well in every 10 min during the 72-hour-long treatment using the zenCellowl incubator microscope (innoME, Espelkamp, Germany). Analyses of cell migration and division as well as a detailed description of scratch assay experiments can be found in Supplementary Materials and Methods.

### Immunofluorescence imaging

SW1573 cells were seeded at 8000 cell/well density on an 8-well chamber slide (Thermo Fisher Scientific, Nunc™ Lab-Tek™ Chamber Slide System, Cat.no: 177402). The next day they were treated with 500 nM tipifarnib, 100 nM sotorasib or their combination for 48 h, then fixed with 4% formaldehyde for 15 min. Cells were permeabilized with 0.1% Triton-X100 for 10 min, washed with DPBS and incubated with *lamin A/C* primary antibody (4777 T, Cell Signaling) dissolved in DPBS (1:200) for an hour at RT. Cells were repeatedly washed with DPBS and incubated with Alexa488 conjugated secondary antibody (Abcam, ab150113) dissolved in DPBS (1:1000) for half an hour at RT. Cells were washed again with DPBS and covered with Vectashield Antifade Mounting Media containing DAPI. For imaging we used a Leica DM RXA epifluorescence microscope (Leica Microsystems, Wetzlar, Germany) equipped with DAPI (excitation 355–425 nm, emission >470 nm, Leica Microsystems, Wetzlar, Germany), Spectrum Green (excitation 460–500 nm, emission 512–542 nm, Vysis, Downers Grove, IL, USA) filter sets for blue and green, respectively. Image acquisition was performed using a Leica DFC365 FX camera and Leica CW4000 FISH software. Two independent experiments were performed for lamin immunofluorescence imaging.

### Calculation of drug interaction, IC50 values and statistics

Drug combinational interactions were determined based on the combinational index theorem calculating Combinational Index (CI) values by CompuSyn Software [30] (https://www.combosyn.com/). This method allows for quantification of drug-drug interactions. CI values less than 1 indicate synergy while values equal to or more than 1 represent additive or antagonistic effect, respectively.

IC50 values in *HRAS* siRNA experiments were calculated by GraphPad Prism 5 software. Normalized viability data from three independent experiments were combined and averaged. Inhibitor concentrations were transformed with the log [10] formula and IC50 data was calculated from the averaged data with the nonlinear regression function of the software.

Statistical analyses were performed in GraphPad Prism 5 software. Normal distribution of data was tested by the Shapiro–Wilk test. Data passing the Shapiro–Wilk test was analyzed by 1way ANOVA or unpaired *t*-test if variance between groups were similar (tested with the Bartlett test before ANOVA or F test before *t*-test), otherwise Kruskal–Wallis or Mann–Whitney *U* test was used. Therefore, an unpaired *t*-test was used for tipifarnib and lonafarnib data from the PRISM Primary Repurposing Screen and GDSC1 screen. Mann–Whitney *U* test was used for the investigation of FTI277 data from the GDSC1 screen. Kruskal–Wallis test followed by Dunn’s Multiple Comparison Test (if the Kruskal–Wallis test was significant) was used for analysis of results from manual counting of cell divisions; changes in G2M phase following treatment using G2M-phase preserving protocol of cell cycle investigation; and data showing changes in length of cytokinesis of SW1573 cells. In animal experiments, the last day’s relative growth values (normalized with each tumor’s starting volumes on the day of the first treatment) and differences in tumor weights were also analyzed by the Kruskal–Wallis test followed by Dunn’s multiple comparison test (*p* < 0.05). Histopathological analyses were tested with ANOVA followed by Tukey post-test for necrosis and mitosis of SW1573 tumors (*p* < 0.05). Otherwise, Kruskal–Wallis test followed by Dunn’s multiple comparison test were applied (*p* < 0.05). For the investigation of *HRAS* and *KRAS* CRISPR dependency from the https://depmap.org database, linear regression was used.

### Supplementary information

Supplementary Figures

Supplementary Materials and Methods

## Data Availability

The datasets generated and/or analyzed during the current study are available from the figshare public repository https://figshare.com/s/d10f0aec047ea9d3beac. Correspondence and requests for materials should be addressed to Balázs Hegedűs.

## References

[1] Prior IA, Hood FE, Hartley JL (2020). The frequency of ras mutations in cancer. Cancer Res.

[2] Timar J, Kashofer K (2020). Molecular epidemiology and diagnostics of KRAS mutations in human cancer. Cancer Metastasis Rev.

[3] Moore AR, Rosenberg SC, McCormick F, Malek S (2020). RAS-targeted therapies: is the undruggable drugged?. Nat Rev Drug Discov.

[4] Ghimessy A, Radeczky P, Laszlo V, Hegedus B, Renyi-Vamos F, Fillinger J (2020). Current therapy of KRAS-mutant lung cancer. Cancer Metastasis Rev.

[5] McCormick F (2020). Sticking it to KRAS: covalent inhibitors enter the clinic. Cancer Cell.

[6] Reck M, Carbone DP, Garassino M, Barlesi F (2021). Targeting KRAS in non-small-cell lung cancer: recent progress and new approaches. Ann Oncol.

[7] Skoulidis F, Li BT, Dy GK, Price TJ, Falchook GS, Wolf J (2021). Sotorasib for lung cancers with KRAS p.G12C mutation. N. Engl J Med.

[8] Dhillon S (2023). Adagrasib: first approval. Drugs.

[9] Nakajima EC, Drezner N, Li X, Mishra-Kalyani PS, Liu Y, Zhao H (2022). FDA approval summary: sotorasib for KRAS G12C-mutated metastatic NSCLC. Clin Cancer Res.

[10] Hong DS, Fakih MG, Strickler JH, Desai J, Durm GA, Shapiro GI (2020). KRAS(G12C) inhibition with sotorasib in advanced solid tumors. N. Engl J Med.

[11] Johnson ML, Ou SHI, Barve M, Rybkin II, Papadopoulos KP, Leal TA (2020). KRYSTAL-1: activity and safety of adagrasib (MRTX849) in patients with colorectal cancer (CRC) and other solid tumors harboring a KRAS G12C mutation. Eur J Cancer.

[12] Timar J (2014). The clinical relevance of KRAS gene mutation in non-small-cell lung cancer. Curr Opin Oncol.

[13] Timar J, Hegedus B, Raso E (2010). KRAS mutation testing of colorectal cancer for Anti-EGFR therapy: dogmas versus evidence. Curr Cancer Drug Targets.

[14] Awad MM, Liu S, Rybkin II, Arbour KC, Dilly J, Zhu VW (2021). Acquired resistance to KRAS(G12C) inhibition in cancer. N. Engl J Med.

[15] Ramos P, Bentires-Alj M (2015). Mechanism-based cancer therapy: resistance to therapy, therapy for resistance. Oncogene.

[16] Ho CSL, Tuns AI, Schildhaus HU, Wiesweg M, Gruner BM, Hegedus B (2021). HER2 mediates clinical resistance to the KRAS(G12C) inhibitor sotorasib, which is overcome by co-targeting SHP2. Eur J Cancer.

[17] Ryan MB, Coker O, Sorokin A, Fella K, Barnes H, Wong E (2022). KRAS(G12C)-independent feedback activation of wild-type RAS constrains KRAS(G12C) inhibitor efficacy. Cell Rep.

[18] Tang D, Kroemer G, Kang R (2021). Oncogenic KRAS blockade therapy: renewed enthusiasm and persistent challenges. Mol Cancer.

[19] Ciardiello D, Maiorano BA, Martinelli E (2022). Targeting KRAS(G12C) in colorectal cancer: the beginning of a new era. ESMO Open.

[20] Yaeger R, Weiss J, Pelster MS, Spira AI, Barve M, Ou SI (2023). Adagrasib with or without Cetuximab in Colorectal Cancer with Mutated KRAS G12C. N. Engl J Med.

[21] Kuboki Y, Yaeger R, Fakih M, Strickler JH, Masuishi T, Kim EJH (2022). 45MO Sotorasib in combination with panitumumab in refractory KRAS G12C-mutated colorectal cancer: Safety and efficacy for phase Ib full expansion cohort. Ann Oncol.

[22] Gibbs JB, Graham SL, Hartman GD, Koblan KS, Kohl NE, Omer CA (1997). Farnesyltransferase inhibitors versus Ras inhibitors. Curr Opin Chem Biol.

[23] Baranyi M, Buday L, Hegedus B (2020). K-Ras prenylation as a potential anticancer target. Cancer Metastasis Rev.

[24] Whyte DB, Kirschmeier P, Hockenberry TN, NunezOliva I, James L, Catino JJ (1997). K- and N-Ras are geranylgeranylated in cells treated with farnesyl protein transferase inhibitors. J Biol Chem.

[25] Ho AL, Brana I, Haddad R, Bauman J, Bible K, Oosting S (2021). Tipifarnib in head and neck squamous cell carcinoma With HRAS mutations. J Clin Oncol.

[26] Lee HW, Sa JK, Gualberto A, Scholz C, Sung HH, Jeong BC (2020). A phase II trial of tipifarnib for patients with previously treated, metastatic urothelial carcinoma harboring HRAS mutations. Clin Cancer Res.

[27] Suzuki M, Jeng LJB, Chefo S, Wang Y, Price D, Li X (2023). FDA approval summary for lonafarnib (Zokinvy) for the treatment of Hutchinson-Gilford progeria syndrome and processing-deficient progeroid laminopathies. Genet Med.

[28] Corsello SM, Nagari RT, Spangler RD, Rossen J, Kocak M, Bryan JG (2020). Discovering the anti-cancer potential of non-oncology drugs by systematic viability profiling. Nat Cancer.

[29] Kwan AK, Piazza GA, Keeton AB, Leite CA (2022). The path to the clinic: a comprehensive review on direct KRAS(G12C) inhibitors. J Exp Clin Cancer Res.

[30] Chou TC (2010). Drug combination studies and their synergy quantification using the Chou-Talalay method. Cancer Res.

[31] Coleman N, Marcelo KL, Hopkins JF, Khan NI, Du R, Hong L (2023). HRAS mutations define a distinct subgroup in head and neck squamous cell carcinoma. JCO Precis Oncol.

[32] Punekar SR, Velcheti V, Neel BG, Wong KK (2022). The current state of the art and future trends in RAS-targeted cancer therapies. Nat Rev Clin Oncol.

[33] Young A, Lou D, McCormick F (2013). Oncogenic and wild-type Ras play divergent roles in the regulation of mitogen-activated protein kinase signaling. Cancer Discov.

[34] Amodio V, Yaeger R, Arcella P, Cancelliere C, Lamba S, Lorenzato A (2020). EGFR blockade reverts resistance to KRAS(G12C) inhibition in colorectal cancer. Cancer Discov.

[35] Ryan MB, Fece de la Cruz F, Phat S, Myers DT, Wong E, Shahzade HA (2020). Vertical pathway inhibition overcomes adaptive feedback resistance to KRAS(G12C) inhibition. Clin Cancer Res.

[36] Misale S, Fatherree JP, Cortez E, Li C, Bilton S, Timonina D (2019). KRAS G12C NSCLC models are sensitive to direct targeting of KRAS in Combination with PI3K Inhibition. Clin Cancer Res.

[37] Brown WS, McDonald PC, Nemirovsky O, Awrey S, Chafe SC, Schaeffer DF (2020). Overcoming adaptive resistance to KRAS and MEK inhibitors by co-targeting mTORC1/2 complexes in pancreatic cancer. Cell Rep. Med.

[38] Hallin J, Engstrom LD, Hargis L, Calinisan A, Aranda R, Briere DM (2020). The KRAS(G12C) inhibitor MRTX849 provides insight toward therapeutic susceptibility of KRAS-mutant cancers in mouse models and patients. Cancer Discov.

[39] Canon J, Rex K, Saiki AY, Mohr C, Cooke K, Bagal D (2019). The clinical KRAS(G12C) inhibitor AMG 510 drives anti-tumour immunity. Nature.

[40] Briere DM, Li S, Calinisan A, Sudhakar N, Aranda R, Hargis L (2021). The KRAS(G12C) inhibitor MRTX849 reconditions the tumor immune microenvironment and sensitizes tumors to checkpoint inhibitor therapy. Mol Cancer Ther.

[41] Moghadamchargari Z, Huddleston J, Shirzadeh M, Zheng X, Clemmer DE, M Raushel F (2019). Intrinsic GTPase activity of K-RAS monitored by native mass spectrometry. Biochemistry.

[42] Javaid S, Schaefer A, Goodwin CM, Nguyen VV, Massey FL, Pierobon M (2022). Concurrent inhibition of ERK and farnesyltransferase suppresses the growth of HRAS mutant head and neck squamous cell carcinoma. Mol Cancer Ther.

[43] Untch BR, Dos Anjos V, Garcia-Rendueles MER, Knauf JA, Krishnamoorthy GP, Saqcena M (2018). Tipifarnib inhibits HRAS-driven dedifferentiated thyroid cancers. Cancer Res.

[44] Adachi Y, Ito K, Hayashi Y, Kimura R, Tan TZ, Yamaguchi R (2020). Epithelial-to-mesenchymal transition is a cause of both intrinsic and acquired resistance to KRAS G12C inhibitor in KRAS G12C-mutant non-small cell lung cancer. Clin Cancer Res.

[45] Janne PA, Riely GJ, Gadgeel SM, Heist RS, Ou SI, Pacheco JM (2022). Adagrasib in non-small-cell lung cancer harboring a KRAS(G12C) mutation. N. Engl J Med.

[46] Zujewski J, Horak ID, Bol CJ, Woestenborghs R, Bowden C, End DW (2000). Phase I and pharmacokinetic study of farnesyl protein transferase inhibitor R115777 in advanced cancer. J Clin Oncol.

[47] Song SY, Meszoely IM, Coffey RJ, Pietenpol JA, Leach SD (2000). K-Ras-independent effects of the farnesyl transferase inhibitor L-744,832 on cyclin B1/Cdc2 kinase activity, G2/M cell cycle progression and apoptosis in human pancreatic ductal adenocarcinoma cells. Neoplasia.

[48] Ashar HR, James L, Gray K, Carr D, McGuirk M, Maxwell E (2001). The farnesyl transferase inhibitor SCH 66336 induces a G(2) -> M or G(1) pause in sensitive human tumor cell lines. Exp Cell Res.

[49] Kelsey I, Manning BD (2013). mTORC1 status dictates tumor response to targeted therapeutics. Sci Signal.

[50] Zheng Y, Xia Y, Hawke D, Halle M, Tremblay ML, Gao X (2009). FAK phosphorylation by ERK primes ras-induced tyrosine dephosphorylation of FAK mediated by PIN1 and PTP-PEST. Mol Cell.

[51] Moudgil DK, Westcott N, Famulski JK, Patel K, Macdonald D, Hang H (2015). A novel role of farnesylation in targeting a mitotic checkpoint protein, human Spindly, to kinetochores. J Cell Biol.

[52] Dubik N, Mai S (2020). Lamin A/C: function in normal and tumor cells. Cancers (Basel).

[53] Adam SA, Butin-Israeli V, Cleland MM, Shimi T, Goldman RD (2013). Disruption of lamin B1 and lamin B2 processing and localization by farnesyltransferase inhibitors. Nucleus.

[54] Strickler JH, Satake H, George TJ, Yaeger R, Hollebecque A, Garrido-Laguna I (2023). Sotorasib in KRAS p.G12C-mutated advanced pancreatic cancer. N. Engl J Med.

[55] Ivanov DP, Parker TL, Walker DA, Alexander C, Ashford MB, Gellert PR (2014). Multiplexing spheroid volume, resazurin and acid phosphatase viability assays for high-throughput screening of tumour spheroids and stem cell neurospheres. PLoS One.

