# Supplementary material for: Farnesyl-transferase inhibitors show synergistic anticancer effects in combination with novel KRAS-G12C inhibitors
Source: Br J Cancer. 2024 Jan 26;130(6):1059–72. doi: 10.1038/s41416-024-02586-x (PMC10951297; doi:10.1038/s41416-024-02586-x)
Supplement: Supplementary file 1 — Supplementary Figures [file 41416_2024_2586_MOESM1_ESM.docx]

**SUPPLEMENTARY FIGURES**

**
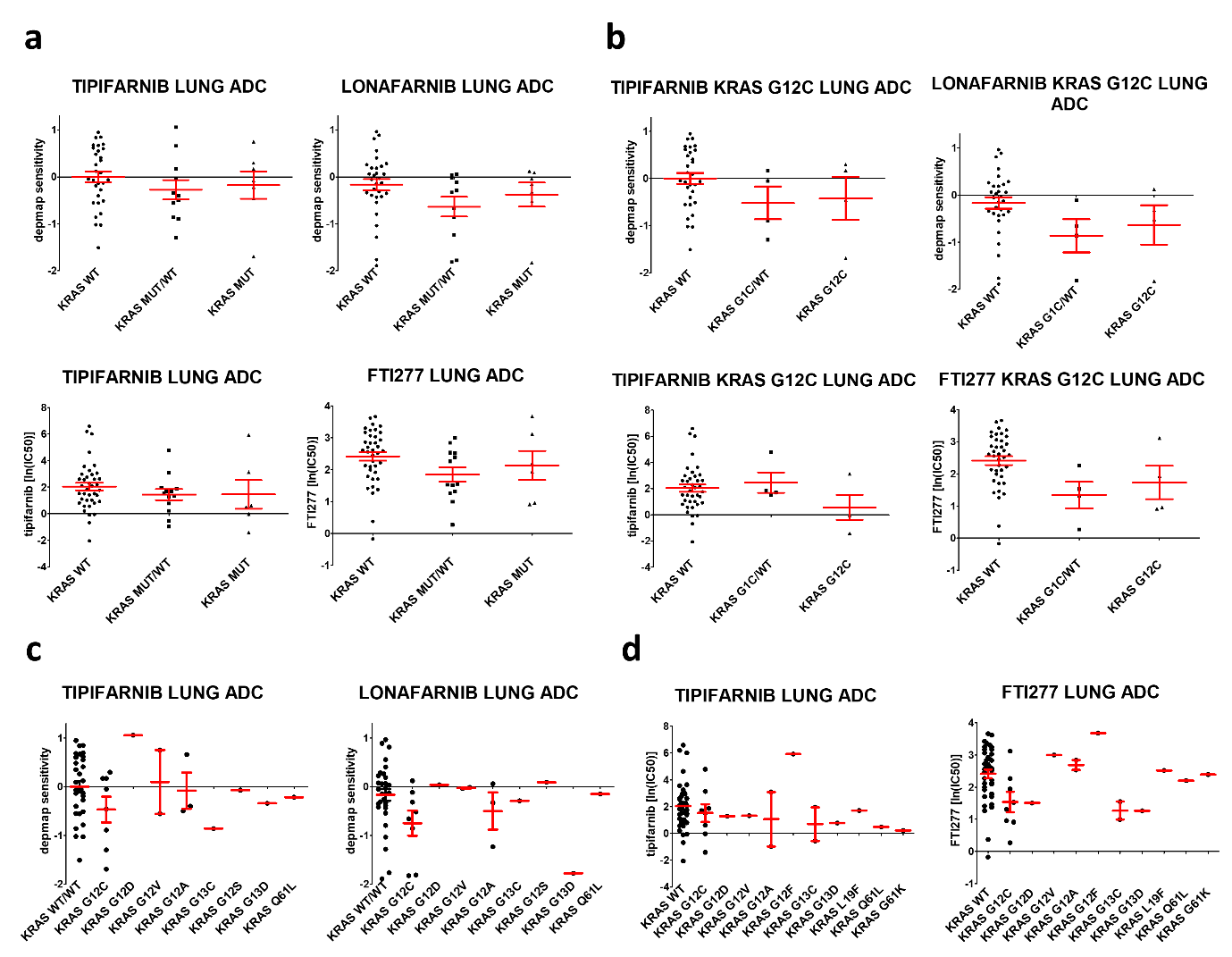
**

**Supplementary Figure 1. Sensitivity of lung adenocarcinoma cell lines to farnesyl-transferase inhibitors based on KRAS mutational type and zygosity. a** Sensitivity of LUAD cell lines to farnesyl-transferase inhibitors based on zygosity of KRAS mutations (WT= KRAS wild type cells, MUT/WT= cells with heterozygous KRAS mutations, MUT= cells with homozygous KRAS mutations). Graphs above include data from PRISM Primary Repurposing Screen and show pooled and normalized (to viability of all 572 or 562 cell lines tested in the screen) sensitivity values to tipifarnib and lonafarnib. Error bars represent SEM for 31 WT KRAS and 11 MUT/WT and 7 MUT cells. Graphs below include data from GDSC1 screen (Genomics of Drug Sensitivity in Cancer from cancerrxgene.org) and show natural logarithm of IC50 to tipifarnib and FTI277. Error bars represent SEM for 39 WT KRAS and 13 MUT/WT and 6 MUT cells. **b** Sensitivity of KRAS G12C mutant LUAD cell lines to farnesyl-transferase inhibitors based on zygosity of KRAS mutations (WT= KRAS wild type cells, G12C/WT= cells with heterozygous KRAS G12C mutations, G12C= cells with homozygous KRAS G12C mutations) Graphs above include data from PRISM Primary Repurposing Screen and show pooled and normalized sensitivity values to tipifarnib and lonafarnib. Error bars represent SEM for 31 WT KRAS and 4 G12C/WT and 4 G12C cells. Graphs below include data from GDSC1 screen and show natural logarithm of IC50 to tipifarnib and FTI277. Error bars represent SEM for 39 WT KRAS and 4 G12C/WT and 4 G12C cells. **c-d** Sensitivity of LUAD cell lines with various types of KRAS mutations to farnesyl-transferase inhibitors. **c** Data from PRISM Primary Repurposing Screen shows pooled and normalized sensitivity values to tipifarnib and lonafarnib. Error bars represent SEM. **d** Data from GDSC1 screen shows natural logarithm of IC50 to tipifarnib and FTI277. Error bars represent SEM. Statistical significance was tested with Kruskal-Wallis test followed by Dunn’s multiple comparison test.

**
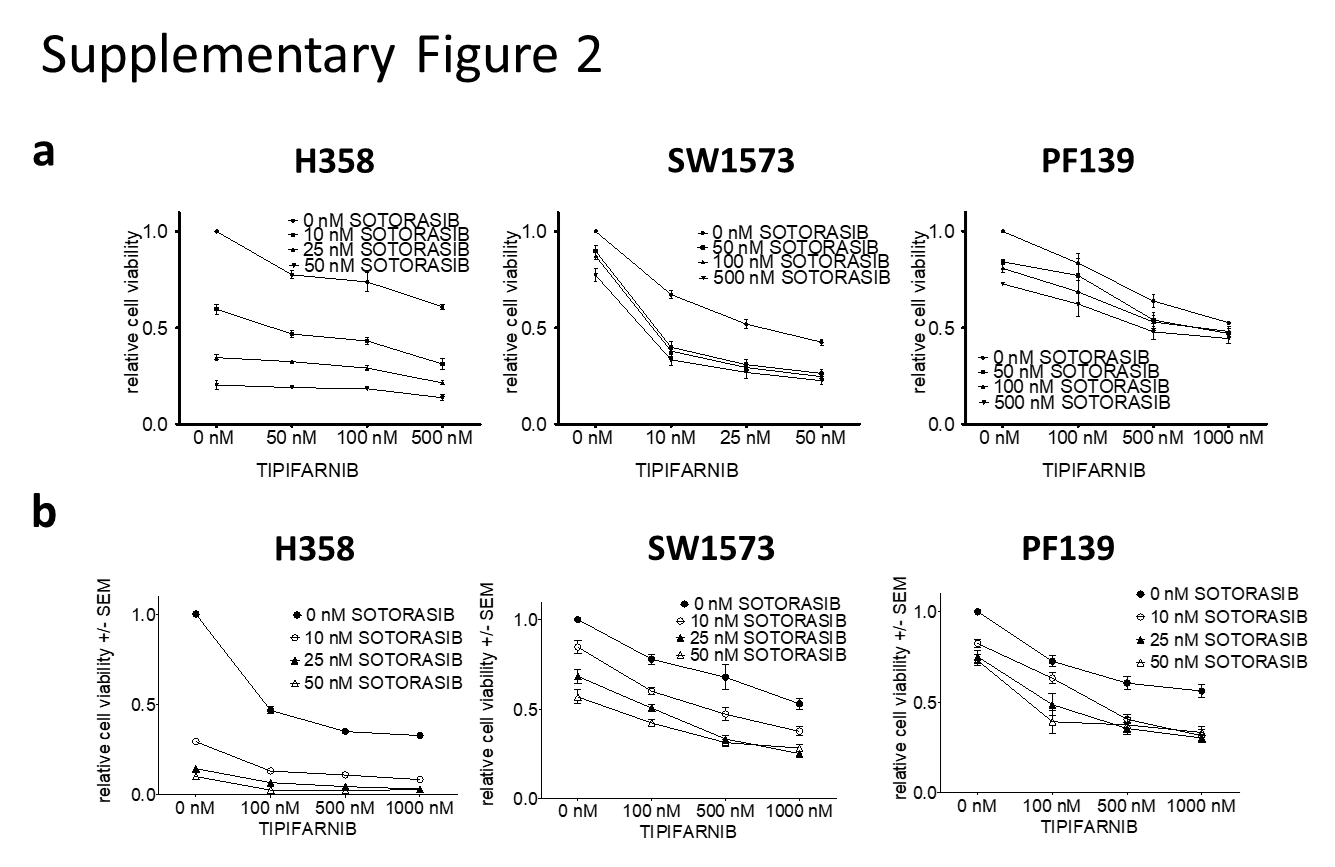
**

**Supplementary Figure 2.** **Cell viability in 2D and 3D combinationsof sotorasib and tipifarnib. a** Control-normalized relative cell viability values derived from 6-day-long 2D SRB tests of H358, PF139 and SW1573 lung adenocarcinoma cells shown in **Figure 2a-b**. **b** Control-normalized spheroid volume data derived from 6-day-long 3D spheroid tests of H358, PF139 and SW1573 lung adenocarcinoma cells (**Figure 2c-e**). Graphs show mean with error bars representing SEM from three independent experiments.

**
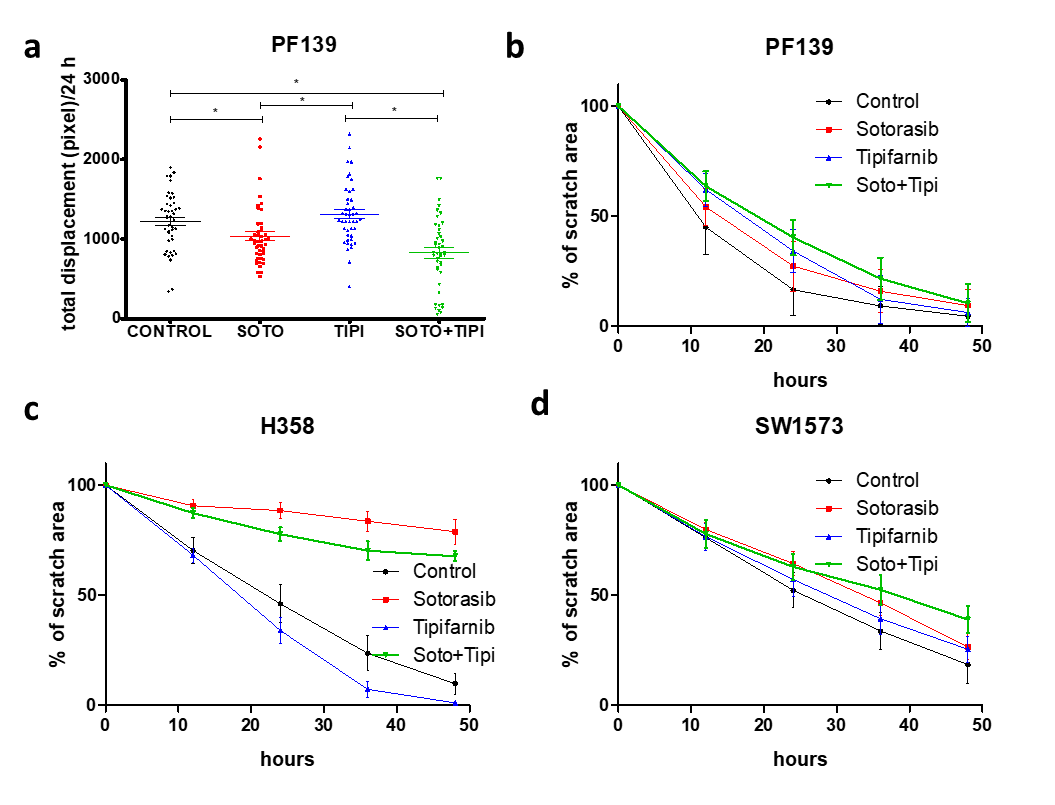
**

**Supplementary Figure 3. Motility of lung adenocarcinoma cells upon sotorasib, tipifarnib and combinational treatment. a** Videomicroscopy analysis of individual cell migration. Total displacement of PF139 cells was analyzed in a 72-hours long treatment with 100 nM sotorasib, 500 nM tipifarnib or their combination between the 24th and the 48th hours. Each treatment was performed in triplicates and total migratory length of 5 randomly chosen cells from each well was measured. Only cells that stayed in the field of view in all frames was included in the analyses Asterisks marks statistically significant differences with p<0.05. Statistical significance was tested with Kruskal-Wallis test followed by Dunn’s multiple comparison test. **b-d**.Scratch assay for PF139 cells following 72-hours long treatment with 100 nM sotorasib, 500 nM tipifarnib or their combination. Percentage of wound area compared to initial total scratch area (of the whole field of view). Results of scratch assay for PF139, H358 and SW1573 cells following 72-hours long treatment with 100 nM sotorasib, 500 nM tipifarnib or their combination. Percentage of wound area compared to initial scratch area (of the whole field of view). Graphs show mean data with error bars representing SEM from three independent experiments.

**
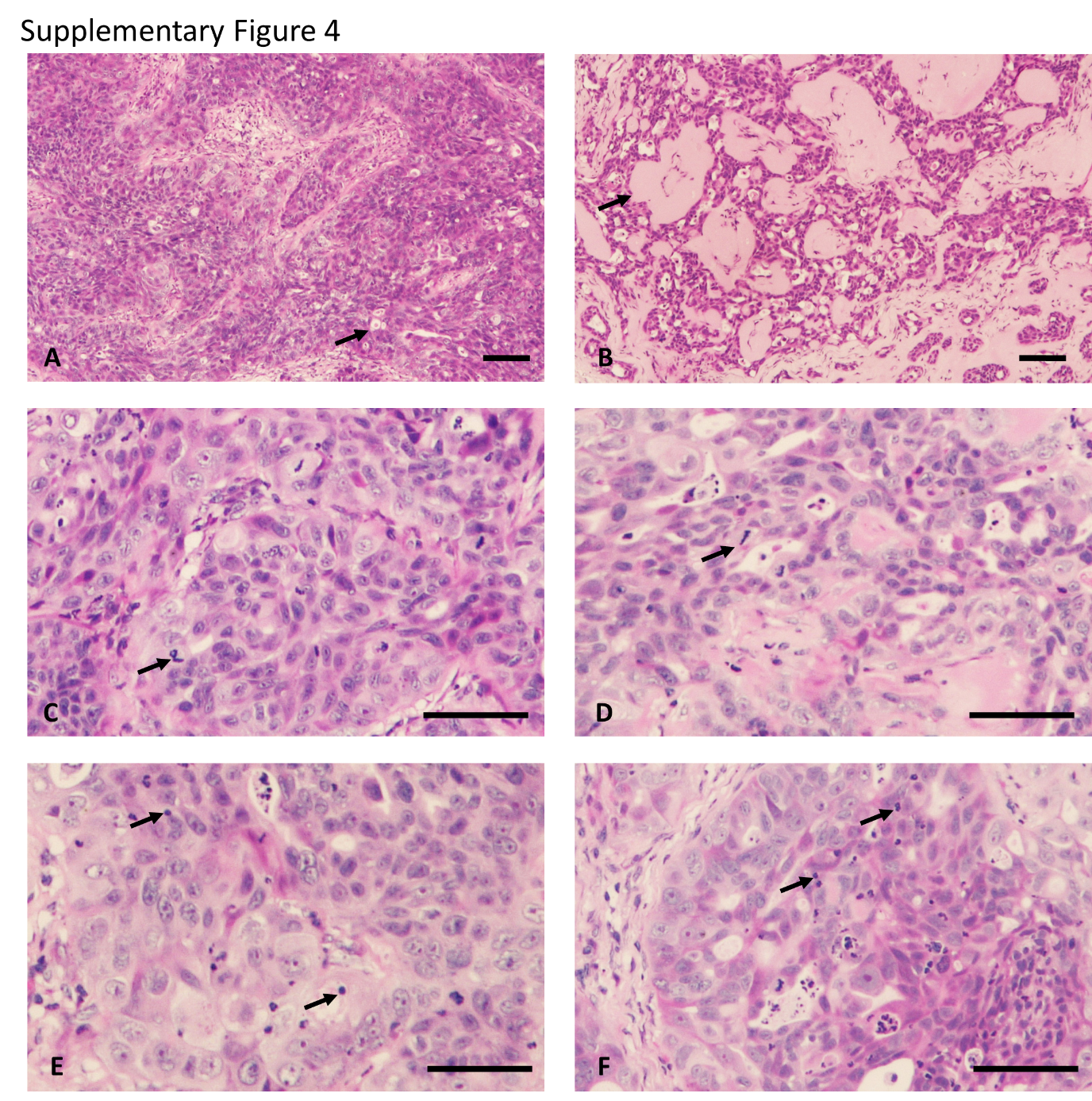
**

**Supplementary Figure 4**. Histological evaluation of H358 tumor xenograft. **A** Necrosis in control tumor. Note the complete absence of confluent necrosis and the presence of single cellular „drop-out” of necrotic tumor cells (arrow). **B** Necrosis in sotorasib and tipifarnib treated H358 tumor. Note the necrotic areas in the tumor tissue filled with serous material (arrow). **C** Mitosis in control tumor. Note the frequent incidence of mitotic figures (arrow). **D** Mitosis in tipifarnib treated H358 tumor. Note the rare occurrence of mitotic figure in the tumor tissue. **E** Apoptosis in control tumor. Note the relatively frequent occurrence of pycnotic, cytoplasm-less apoptotic bodies in the tumor tissue (arrow). **F** Tipifarnib treated tumor. Note the frequent occurrence of apoptotic bodies in the tumor tissue (arrow). H&E stainings. Bars correspond to 250 µm.

**
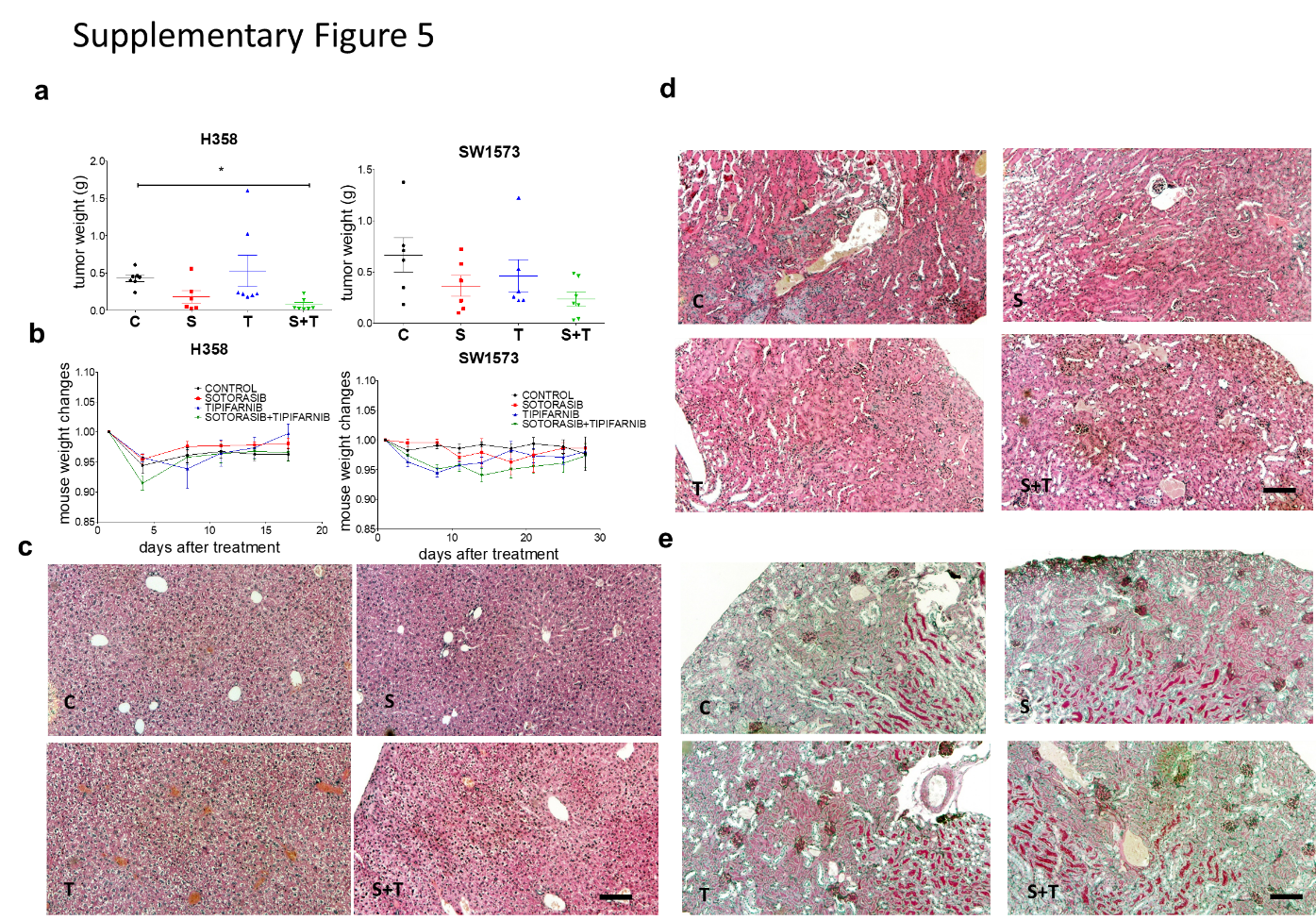
**

**Supplementary Figure 5. Changes in body and tumor weight upon *in vivo* treatment with sotorasib, tipifarnib or their combination.**. **a** Final tumor weights after resection. Error bars represent SEM. In H358 xenografts, only combinational treatment resulted in significantly smaller tumors compared to control (p=0.0028), while differences in SW1573 tumors did not reach statistical significance (p=0.122). Asterisks marks statistically significant differences with p<0.05. Statistical significance was tested with Kruskal-Wallis test followed by Dunn’s multiple comparison test. **b** Bodyweight losses did not reach 10% during the study and were around at maximum of 5% during the investigation. **c** Histology of the liver after treatment by 25 mg/kg sotorasib, 40 mg/kg tipifarnib or their combination. C=control, S=sotorasib. T=tipifarnib. S+T=combinational therapy. Note the intact liver morphology after all type of treatments. H&E staining. Bars are 250 µm. **d-e** Histological analyses of the kidneys by H&E (**d**) and PAS (**e**) staining after treatment by, 25 mg/kg sotorasib, 40 mg/kg tipifarnib or their combination. Note the intact kidney morphology after all type of treatments. Bars indicate 250 µm.


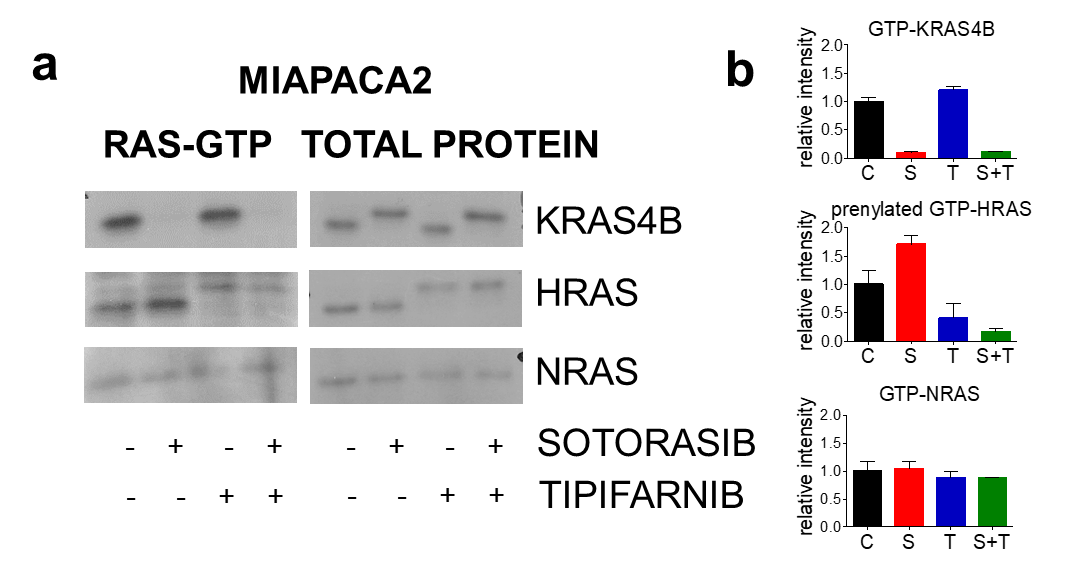


**Supplementary Figure 6. RAS protein levels and activation following 48-hour-long treatment with sotorasib (100 nM) and/or tipifarnib (500 nM).** **a** Images show representative blots of the RAS proteins. RAS-GTP stands for only GTP-bound, active RAS proteins while total protein shows blots from the whole cell lysates. **b** Graphs represent normalized level of GTP-bound KRAS4B, HRAS and NRAS, respectively. Note that graph shows only changes of prenylated fraction of HRAS. Protein levels were normalized to control. Graphs show mean data with error bars representing SEM from three independent experiments.


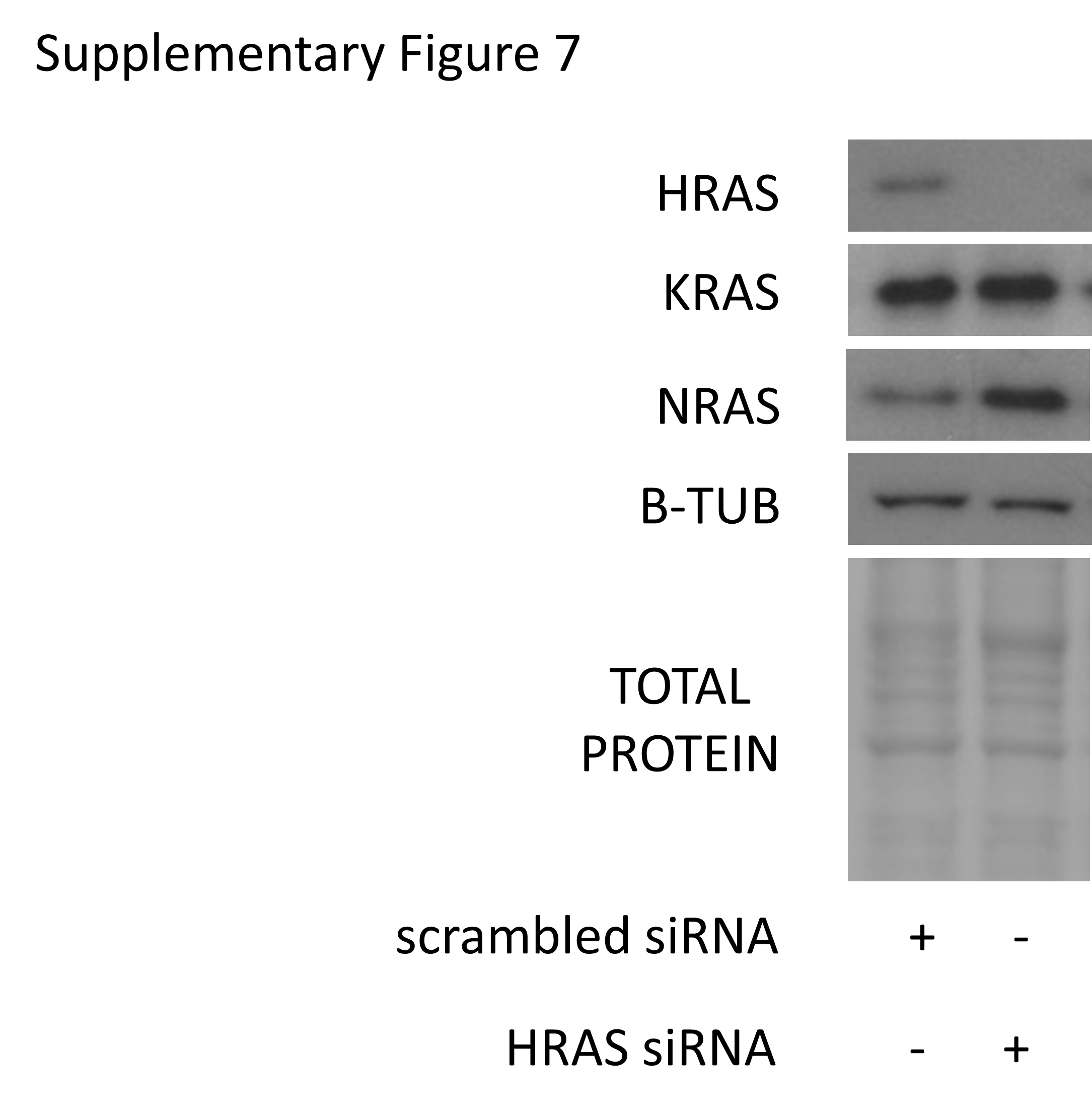


**Supplementary Figure 7. Validation of successful and specific siRNA-mediated HRAS knockdown in IC50 experiments shown in Figure 6.** Images show representative blots of the specified proteins from three independent experiments with SW1573 isolated after 6-days exposure to non-targeting and HRAS specific siRNA. Level of HRAS was successfully reduced by HRAS siRNA while KRAS protein level was not changed. A compensatory increase in NRAS level can also be observed.

**
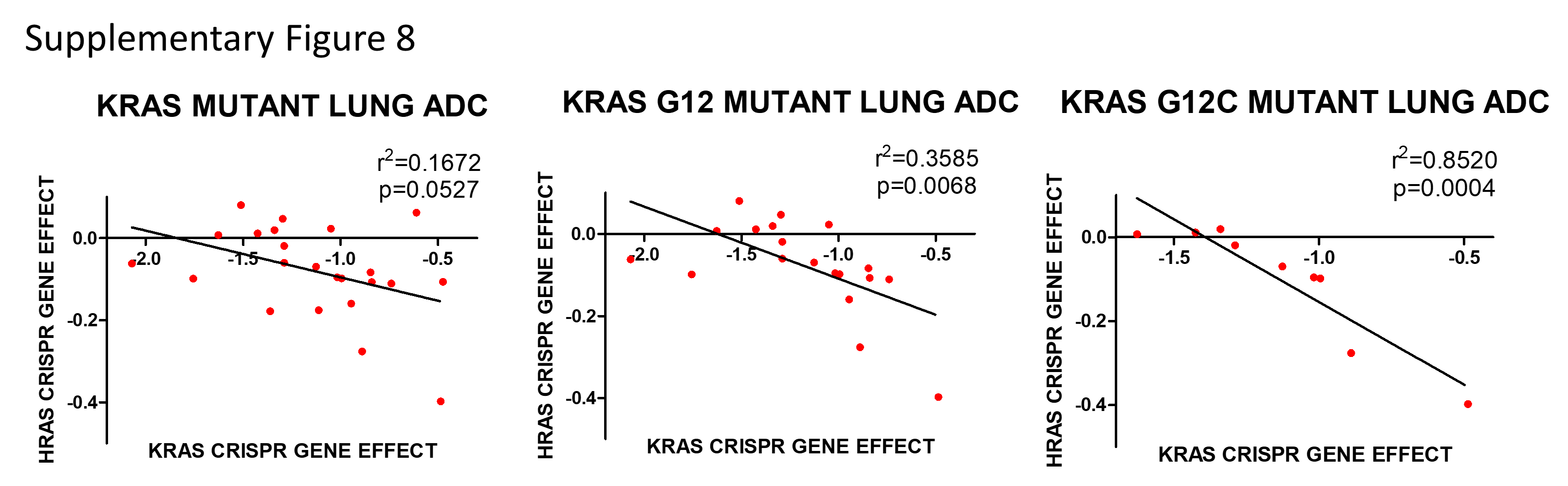
**

**Supplementary Figure 8. Relation of CRISPR dependency of KRAS mutant lung adenocarcinoma cells on HRAS and KRAS genes.** Results show CRISPR sensitivity data (DepMap 22Q2 Public+Score, Chronos) obtained from depmap.org. Filters were set for NSCLC adenocarcinoma cell lines and combined CRISPR sensitivity values were downloaded. The type of KRAS mutation was determined for each cell line based on cellosaurus.org. Lung adenocarcinoma cell lines with mutation on KRAS codon G12 show significant negative correlation between HRAS and KRAS dependency. This correlation is even stronger when only KRAS-G12C mutant cell lines are included.

**
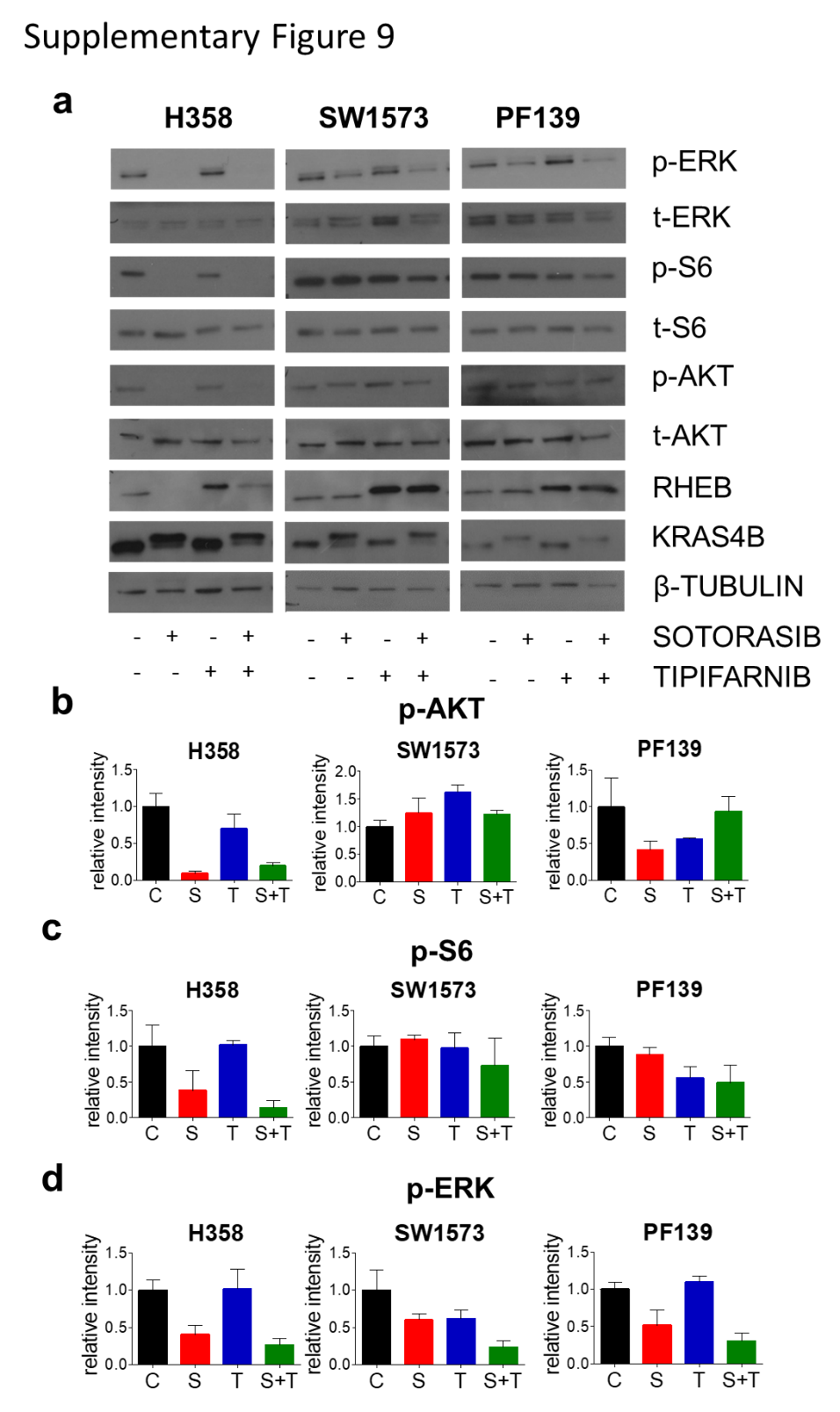
**

**Supplementary Figure 9. RAS mediated signaling networks and changes in RAS protein expression and activation following 48-hour-long treatment with sotorasib (100 nM) and/or tipifarnib (500 nM)** **a** Images show representative blots of the specified proteins. **b-d** Graphs represent normalized level of activated Akt, S6 and ERK1/2 respectively. Protein levels were normalized to total protein (Ponceau) and to control. Activation of downstream elements of RAS signaling pathways show marked but cell line specific changes after treatments.

**
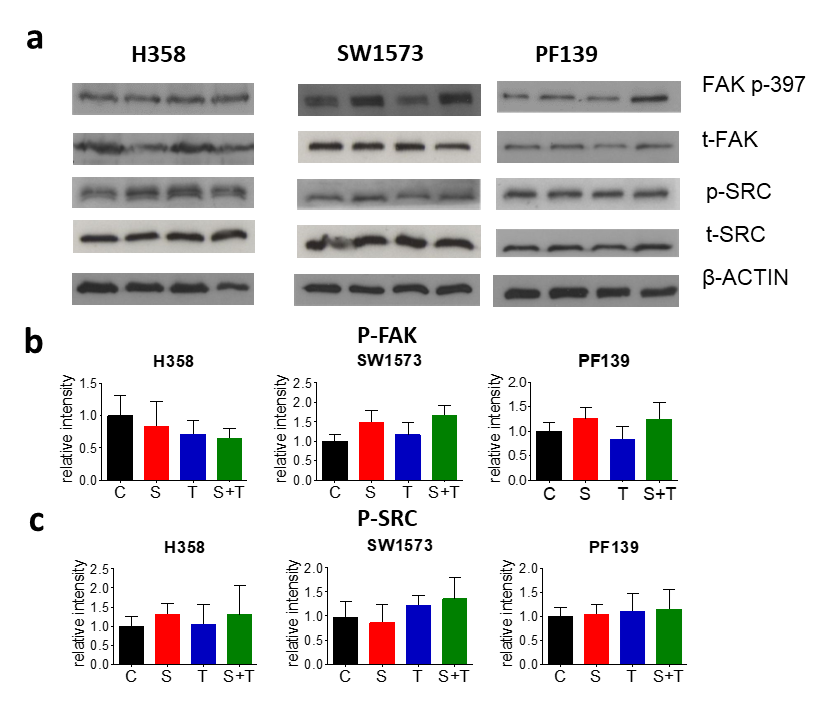
**

**Supplementary Figure 10. Changes in activation of FAK-SRC signaling pathway following 48-hour-long treatment with sotorasib (100 nM) and/or tipifarnib (500 nM) a** Images show representative blots of the specified proteins. **b-c** Graphs represent normalized level of activated FAK (**b**) and SRC (**c**). Protein levels were normalized to total protein (Ponceau) and to control. Activation of SRC showed minor changes upon treatments. Autophosphorylation of FAK was increased upon sotorasib and combinational treatment in SW1573 and PF139 cell lines. In H358, all treatment reduced level of p-FAK.


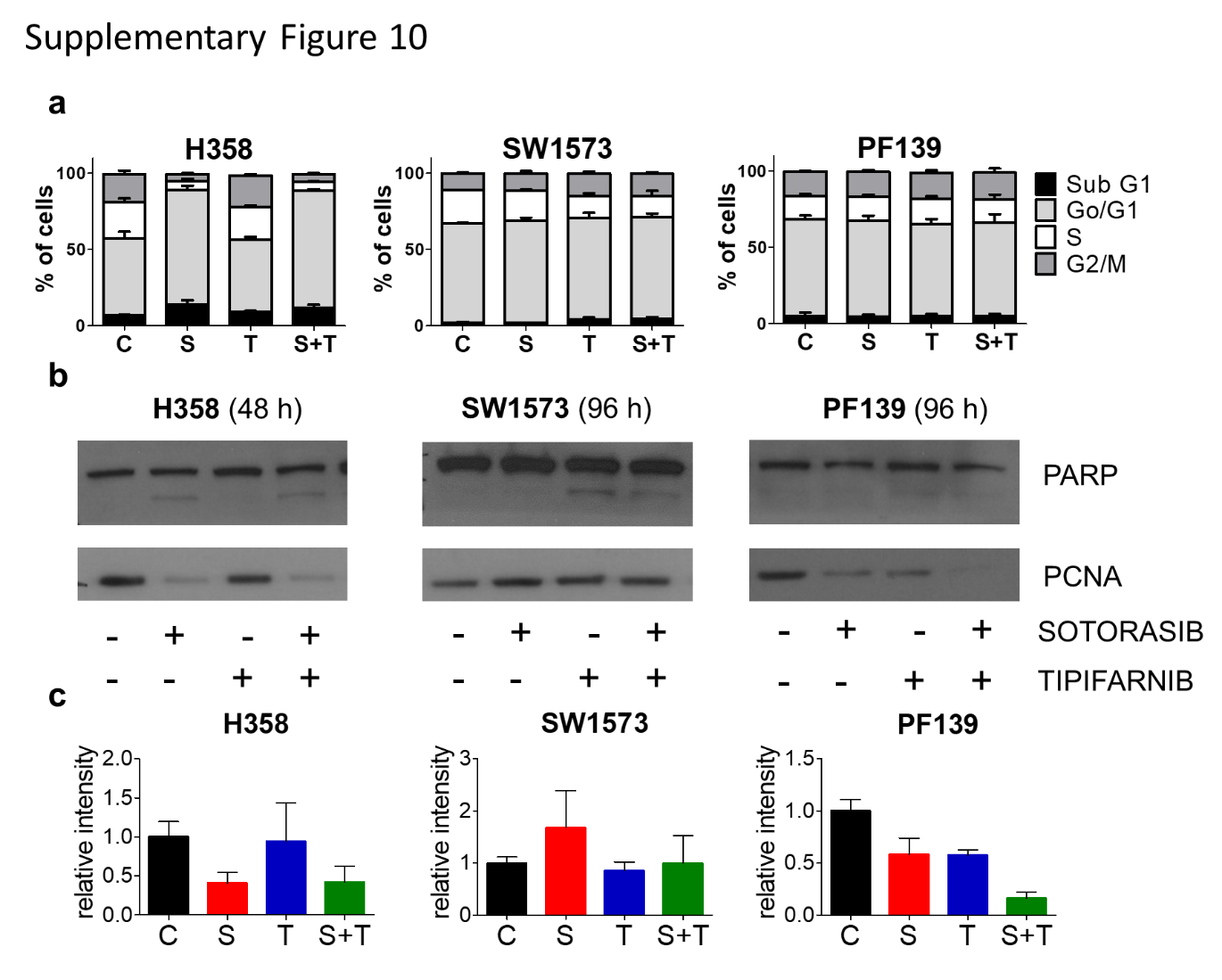


**Supplementary Figure 11. Changes in cell cycle distribution and apoptosis and proliferation markers upon sotorasib and/or tipifarnib treatment.** Cells were treated with 100 nM sotorasib and/or 500 nM tipifarnib for 96 hours (or 48 hours in case of H358) **a** Changes in cell cycle distribution. Tipifarnib induced accumulation of G2/M and subG1 phase cells in H358 and SW1573. Sotorasib and combinational treatment drastically increased the ratio of the cells in the subG1 (apoptotic cells) and strongly reduced it in the G2M and S G0/1 phases. **b** Western blot analyses of apoptosis marker PARP and proliferation marker PCNA. H358 and SW1573 show apoptosis induction upon both single and combinational treatments. **c** PCNA level decreased in H358 and PF139 cell lines upon all treatments. Interestingly, no detectable change or even increased level of PCNA could be observed in sotorasib resistant SW1573 cell line. All data shown are from three independent experiments. Graphs show mean with error bars representing SEM.

**
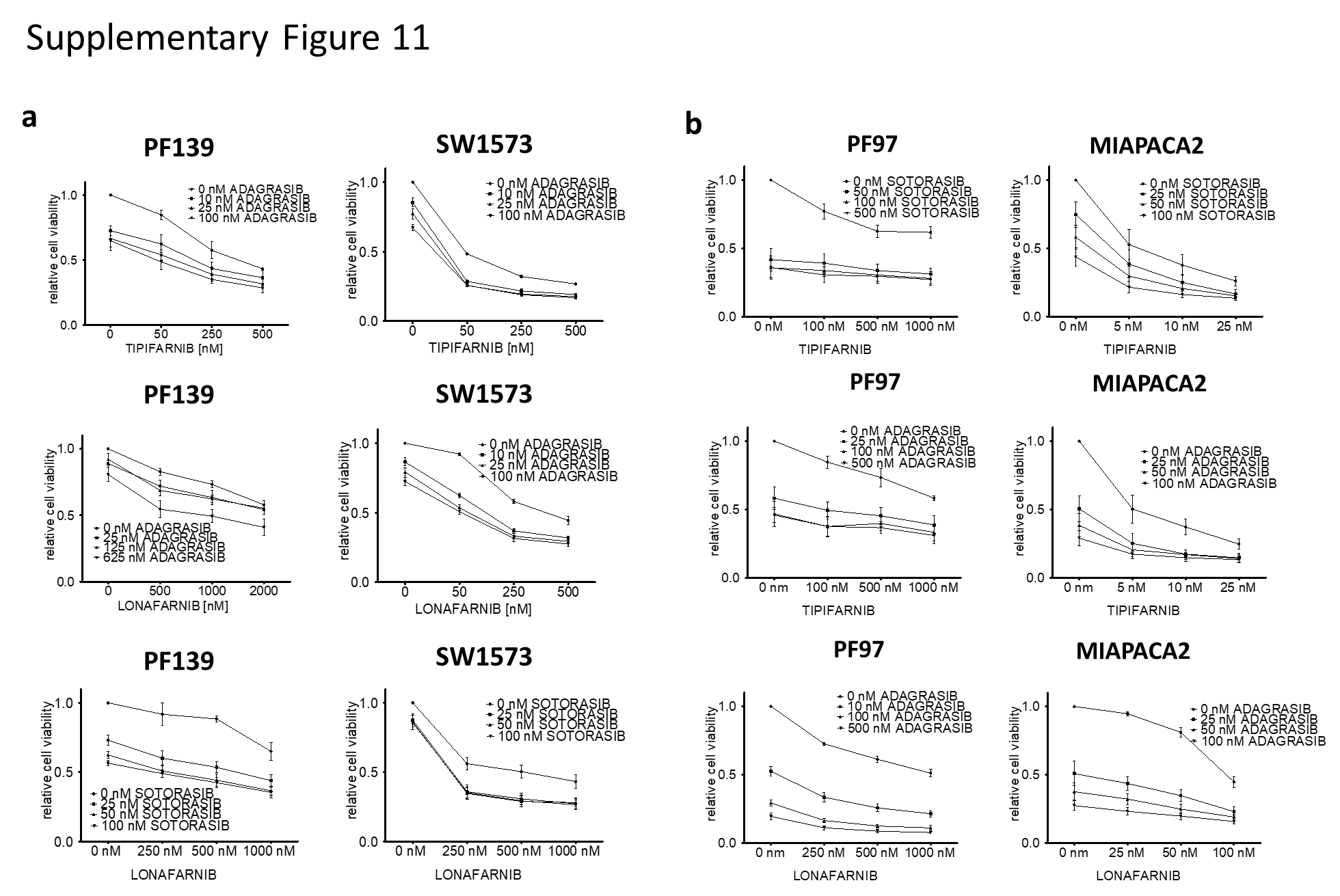
**

**Supplementary Figure 12. Results from 2D combinational tests showing different combinational settings of sotorasib, tipifarnib, adagrasib and lonafarnib. a** Control-normalized relative cell viability values derived from 6-day-long 2D SRB tests of PF139 and SW1573 lung adenocarcinoma cell lines shown in **Figure 6 a-b**.**b** Control-normalized relative cell viability values derived from 6-day-long 2D SRB tests of PF97 colorectal and MIAPACA2 pancreatic adenocarcinoma cell lines shown in **Figure 6 c-d** Graphs show mean data with error bars representing SEM from three independent experiments.

**SUPPLEMENTARY TABLES**

**Supplementary Table 1. Origin and key features of cell lines used in this study**

| **Cell line** | **Age** | **Gender** | **Origin** | ***KRAS*** | ***TP53* status** | **Other** |
| --- | --- | --- | --- | --- | --- | --- |
| **H358** | **n.a.** | **Male** | **lung** | **Heterozygous p.Gly12Cys** | **Homozygous del.** |  |
| **PF139** | **75y** | **Male** | **lung** | **Heterozygous p.Gly12Cys** | **p.Arg282Trp** |  |
| **SW1573** | **44y** | **Female** | **lung** | **Homozygous p.Gly12Cys** |  | **Homozygous del. of *CDKN2A* and *SMAD4*;  Heterozygous *CTNNB1* p.Ser33Phe; Heterozygous *PIK3CA* p.Lys111Glu;**  **Heterozygous *SMARCB1*  c.362+1G>C** |
| **PF97** | **75y** | **Male** | **colorectal** | **Heterozygous p.Gly12Cys** |  | **Heterozygous *PTEN* p.Ala79Thr** |
| **MIAPACA2** | **65y** | **Male** | **pancreas** | **Homozygous p.Gly12Cys** | **Homozygous p.Arg248Trp** | **Homozygous del. of *CDKN2A*** |

**Supplementary Table 2. List of farnesylated proteins based on the UniProt database (www.uniprot.org)**

| **Entry** | **Length** | **Gene names** |
| --- | --- | --- |
| P51157 | 221 | RAB28 |
| P16499 | 860 | PDE6A PDEA |
| Q96MT3 | 831 | PRICKLE1 RILP |
| P10114 | 183 | RAP2A |
| Q15831 | 433 | STK11 LKB1 PJS |
| Q93096 | 173 | PTP4A1 PRL1 PTPCAAX1 |
| P17081 | 205 | RHOQ ARHQ RASL7A TC10 |
| Q96I34 | 528 | PPP1R16A MYPT3 |
| Q12974 | 167 | PTP4A2 PRL2 PTPCAAX2 BM-008 |
| P09936 | 223 | UCHL1 |
| P62070 | 204 | RRAS2 TC21 |
| Q8TAI7 | 183 | RHEBL1 |
| P01111 | 189 | NRAS HRAS1 |
| P01116 | 189 | KRAS KRAS2 RASK2 |
| Q9UP65 | 541 | PLA2G4C |
| O75781 | 387 | PALM KIAA0270 |
| Q9Y272 | 281 | RASD1 AGS1 DEXRAS1 |
| Q9NRR6 | 644 | INPP5E |
| P61587 | 244 | RND3 ARHE RHO8 RHOE |
| P01112 | 189 | HRAS HRAS1 |
| Q9H4E5 | 214 | RHOJ ARHJ RASL7B RHOI TCL |
| P20700 | 586 | LMNB1 LMN2 LMNB |
| O75365 | 173 | PTP4A3 PRL3 |
| O15498 | 198 | YKT6 |
| Q8NFA0 | 1604 | USP32 USP10 |
| P43119 | 386 | PTGIR PRIPR |
| P62745 | 196 | RHOB ARH6 ARHB |
| Q96T49 | 567 | PPP1R16B ANKRD4 KIAA0823 |
| Q7Z7G2 | 160 | CPLX4 |
| Q13286 | 438 | CLN3 BTS |
| P09543 | 421 | CNP |
| P49454 | 3114 | CENPF |
| Q8WW22 | 397 | DNAJA4 |
| O60884 | 412 | DNAJA2 CPR3 HIRIP4 |
| Q02224 | 2701 | CENPE |
| P31689 | 397 | DNAJA1 DNAJ2 HDJ2 HSJ2 HSPF4 |
| P63211 | 74 | GNGT1 |
| P32455 | 592 | GBP1 |
| Q03252 | 620 | LMNB2 LMN2 |
| P32019 | 993 | INPP5B OCRL2 |
| Q9P2W3 | 67 | GNG13 |
| P46020 | 1223 | PHKA1 PHKA |
| P40855 | 299 | PEX19 HK33 PXF OK/SW-cl.22 |
| Q93100 | 1093 | PHKB |
| P46019 | 1235 | PHKA2 PHKLA PYK |
| Q15835 | 563 | GRK1 RHOK |
| Q14642 | 412 | INPP5A 5PTASE |
| Q15382 | 184 | RHEB RHEB2 |
| P55209 | 391 | NAP1L1 NRP |
| P02545 | 664 | LMNA LMN1 |
| Q92737 | 203 | RASL10A RRP22 |
| Q96D21 | 266 | RASD2 TEM2 |
| P61952 | 73 | GNG11 GNGT11 |
| A6NDB9 | 673 | PALM3 |
| O14610 | 69 | GNGT2 GNG8 GNG9 GNGT8 |
| Q8WVH0 | 158 | CPLX3 Nbla11589 |
| Q7Z444 | 233 | ERAS HRAS2 HRASP |
| Q5VW32 | 411 | BROX BROFTI C1orf58 |
| Q7Z3G6 | 844 | PRICKLE2 |
| Q8IXS6 | 379 | PALM2 |
| O15255 | 209 | RTL8C CXX1 FAM127A MAR8 MAR8C MART8 hucep-5 |
| A6NMN0 | 1240 | PHKA1 |
| A6NIT2 | 1181 | PHKA1 |
