# Supplementary material for: Farnesyl-transferase inhibitors show synergistic anticancer effects in combination with novel KRAS-G12C inhibitors
Source: Br J Cancer. 2024 Jan 26;130(6):1059–72. doi: 10.1038/s41416-024-02586-x (PMC10951297; doi:10.1038/s41416-024-02586-x)
Supplement: Supplementary file 2 — Supplementary Materials and Methods [file 41416_2024_2586_MOESM2_ESM.docx]

**Supplementary Materials and Methods:**

**Cell culture, mycoplasma detection and cell authentication**

Cells were cultured in DMEM (Dulbecco’s Modified Eagle Medium) (Lonza, Basel, Switzerland; with 4500 mg/dm3 glucose, pyruvate and L-glutamine) supplemented with 10% fetal bovine serum (FBS (EuroClone, Pero, MI, Italy) and 1% penicillin-streptomycin-amphotericin (Lonza) in tissue culture flasks in a humidified 5% CO2 atmosphere at 37 ºC. Detection of possible Mycoplasma contamination was performed and was found negative by Mycoprobe kit (Bio-Techne). Authentication of H358, PF97 and PF139 was performed by Multiplexion (SNP-based authentication), while SW1573 was authenticated by ATCC (STR-based authentication). Experiments MIAPACA2 was performed with low (below 10) passage number following purchase from ATCC.

**Polyhema plate preparation**

Spheroids were grown in polyHEMA (polyhydroximethylmethylacrylate) coated 96-well round bottom plates (Sarstedt, Nümbrecht, Germany). Briefly, the inner 60 wells of the plate were coated with 60 µl 5 mg/ml polyHEMA dissolved in 96% ethanol. Plates were dried at 60 °C in a plate shaker while shaking, then sterilized with UV.

**Cell cycle assay**

Determination of DNA content in each cell was used to evaluate the number of cells in each cell cycle phase as described earlier.(50) Briefly, 500 nM tipifarnib, 100 nM sotorasib or their combination were used for treatment. All cells were treated for 96 hours in 6-well plates, except for H358, which was only treated for 48 hours due to its higher sensitivity to sotorasib. Cells were trypsinized and lysed before staining with DAPI (4′,6-diamidino-2-phenylindole) for 5 min at 37°C. After adding the stabilization buffer, 10 µl of each sample was loaded onto an 8-well NC slide. NucleoCounter NC-3000™ system (Chemometec, Allerod, Denmark) was used to quantify cellular fluorescence.

Modified M phase preserving protocol was performed by prior fixation of the cell suspension by ice-cold 70% ethanol before lysis.

**siRNA knockdown experiments**

Cells that reached 70-80% confluence were trypsinized and counted using Luna II Automated Cell Counter. Cells were plated in 2500 and 7500 cells/well density (for non-targeting siRNA and HRAS siRNA treatment, respectively) on a 48-well plate and in 25000 cells/well on a 6-well plate (for immunoblot validation sample) supplemented with pre-mixed Lipofectamine RNAiMAX Reagent (Thermo Fisher Scientific) and OptiMEM (Thermo Fisher Scientific) containing 2.5 pmol/well non-targeting or HRAS siRNA according to the manufacturer’s instruction (reverse transfection protocol). Next day, cells on 48-well plates were treated with tipifarnib or sotorasib with 0, 1, 5, 25, 125 and 625 nM final concentration. On the 3rd day of the treatment, cells were supplemented with fresh pre-mixed 2.5 pmol/well siRNA diluted in Lipofectamine RNAiMAX Reagent and OptiMEM according to the manufacturer’s instruction. After 6 days, wells were washed with DPBS (Lonza) and the cells were fixed by 10% trichloroacetic acid and stained with SRB (Sigma) dye for 15 minutes. Plates were then repeatedly washed with 1% acetic acid to remove excess dye. Protein-bound SRB was then dissolved in 10 mM Tris buffer (pH=7,4) and OD was measured at 570 nm using a microplate reader (EL800, BioTec Instruments, Winooski, VT, USA). OD values were normalized to control, and IC50 values were calculated from transformed data using GraphPad Prism 5 software. Immunoblot samples were isolated and investigated to validate successful HRAS knockdown as stated at immunoblot method section.

**Western blotting**

Protein expression and phosphorylation were investigated by immunoblot assays. Following 48 hours (samples for RAS signalization and detection of lamin) or 96 hours (samples for PARP and PCNA) treatment with 500 nM tipifarnib, 100 nM sotorasib or their combination in 6-well plates, cells were washed with DPBS and fixed with 6% trichloroacetic acid for an hour at 4°C. Cells were mechanically harvested and centrifuged at 6000 RCF for 15 minutes. Precipitated protein was dissolved in modified Läemmli-type sample buffer containing 0.02% bromophenol blue, 10% glycerol, 2% SDS, 100 mM dithiothreitol (DTT), 5 mM EDTA, 125 mg/ml urea, 90 mM Tris-HCl, pH 7.9. Qubit fluorometer was used for determination of protein concentration. Equal amounts of protein (20 µg/lane) were loaded onto 10% poliacrylamid gels and transferred after electrophoretic separation to PVDF (polyvinylidene fluoride) membranes. Analyses of RAS mediated signaling were performed using p-AKT (4058S), AKT (9272S), p-S6 (2215S), S6 (2217S), p-ERK1/2 (9101S), ERK1/2 (9102S), RHEB (13879S), FAK (3285), p-FAK (3283) and β-TUBULIN (2128S) primary antibodies (all Cell Signaling Technology, Danvers, MA, USA). For detection of apoptosis and cell proliferation PARP (9545S, Cell Signaling Technology) and PCNA (13110S, Cell Signaling Technology) primary antibody was used, respectively. Detection of changes in nuclear lamina was performed using a lamin A/C primary antibody (4777T, Cell Signaling Technology). All antibodies were dissolved according to the manufacturer’s instructions in 5% BSA or dry milk in 1x TTBS (tris-buffered saline supplemented with 1% Tween80) buffer. Membranes were blocked at room temperature in 5% dry milk dissolved in 1x TTBS for an hour, then were incubated in primary antibodies overnight at 4°C. HSP (horse-radish peroxidase) conjugated rabbit secondary antibodies (1:10000, 1 h, RT) and Pierce ECL Western Blotting Substrate (Thermo Fisher Scientific, Waltham, MA, USA) were used for visualization. Ponceau staining was used for normalization. Quantification was performed using ImageJ software using “sum of all data points in a replicate” method as described in (1). Each cell line was analyzed in 3 biological replicates.

**Active RAS pulldown experiments**

SW1573 cells were cultured in 6-well plates and left for a day to attach to the surface. Next day, the medium was replaced to fresh medium containing the inhibitors. Following 48 hours treatment with 500 nM tipifarnib, 100 nM sotorasib or their combination, cells were isolated according to the manufacturer’s instruction. Briefly, cells were lysed with Lysis/Binding/Wash buffer containing proteinase inhibitor and lysates were centrifuged at 16000xg. Supernatant was transferred to a new Eppendorf tube and protein concentration was determined with Qubit fluorometer. 1200 µg from each sample was then incubated with GST-Raf1-RBD and Glutathione Agarose Resin for an hour at room temperature and was centrifuged in a spin cup. Trapped GTP-bound RAS protein was removed from the spin column and dissolved in 50 µl DTT containing 2X SDS sample buffer. Equal volume was then loaded onto 10% poliacrylamide gels and transferred after electrophoretic separation to PVDF membranes. Signal development was performed as stated in the above section. For detection of KRAS4B WH0003845M1 antibody was purchased from Sigma, HRAS was detected by 18295-1-ap from Proteintech and SC-31 antibody (Santa Cruz Biotechnology, Dallas, TX, USA) was used for NRAS.

**Cell migration analyses**

For cell migration, videos of PF139 cells were analyzed using CellTracker_v1_1_1 Standalone Version free software.(2,3) Cells were seeded into 3 wells for each treatment type. Five cells were randomly chosen from each field of view and their length of displacement was measured using manual tracking from 24 to 48 hours of the treatment. Only cells that stayed in the field of view in all frames (from 144-288) were chosen. Before tracking, videos were processed with the software: frames outside 144-288 were removed, then vignetting correction was applied with bicubic interpolation (block size: 120, maximum allowed displacement: 60). Manual tracking was performed by skipping 5 images with every click, on these frames dynamic interpolation was applied (maximum cell displacement: 30; cell diameter: 65 and using template matching). Tracks were manually reviewed and corrected if necessary. Total displacement data of the tracks was used for later analysis. Experiments were repeated thrice independently and were collected and analyzed together.

**Scratch assay**

Scratch assays were performed in two independent settings. One biological replicate for each LUAD cell line (H358, PF139 and SW1573) was prepared using the MuviCyte™ live-cell Imaging System (PerkinElmer, Waltham, MA) supplied with a MuviCyte™ scratcher (PerkinElmer) to perform standardized scratches on 96 well plates following the manufacturer’s instructions; while two further biological replicates were prepared using the zenCellowl incubator microscope (innoME, Espelkamp, Germany) with 24 well plates preparing scratch manually with a 10 µl pipette tip using a mold provided by the manufacturer. Briefly, cells were seeded at high number to cover confluently the whole well and left for overnight to attach. Next day, scratches were made, wells were washed with DPBS to remove debris and fresh medium were added supplemented with the inhibitors (100 nM sotorasib, 500 nM tipifarnib or their combination). For MuviCyte™ experiments, four parallel wells were assigned to each treatment group, while one well was used per treatment for zenCellowl experiments. Wound closure was monitored for 72 hours pictures taken from every well in every 6^th^ hour. Analyses of video was performed using a modified script of (4) for ImageJ Fiji software. The script measures automatically area of the scratch for different timepoints. Data were normalized to and expressed as percentage of the starting scratch area. For Muvicyte experiments, data from four wells per treatment group were averaged and used as a single replicate graphed with data from zenCellowl experiments in GraphPad Prism 5 software. In summary, three independent biological replicates were included in the analysis. T_1/2_ wound closure data was calculated with GraphPad Prism 5 software using non-linear regression (log[inhibitor] vs normalized response, variable slope).

**Cell division and cytokinesis length analyses**

Cell divisions were counted manually using the Cell Counter plugin of the ImageJ Fiji software. Technically, a grid of 350000 pixels per area were applied to each video and number of divisions were determined in three pre-determined squares. Counted cell divisions in each field of view were summed from 0 to 24th, from 24th to 48th and finally from 48th to 72nd hours of the experiments and evaluated using GraphPad Prism 5 software (GraphPad, La Jolla, San Diego, CA, USA). Each graph shows data from three independent experiments. Determination of length of cytokinesis was performed similarly to cell division counting. Videos were analyzed from the 48th hour after treatment. 350000 pixel2 grid was also applied of which six squares were involved in the analyzes. Length of cytokinesis of the first dividing cell in each square was determined, resulting in six data point/video. Length values were combined and statistically analyzed using GraphPad Prism 5 software.

**References**

1. Degasperi A, Birtwistle MR, Volinsky N, Rauch J, Kolch W, Kholodenko BN. Evaluating strategies to normalise biological replicates of Western blot data. PloS one **2014**;9:e87293

2. Piccinini F, Kiss A, Horvath P. CellTracker (not only) for dummies. Bioinformatics **2016**;32:955-7

3. <http://celltracker.website/index.html>.

4. Suarez-Arnedo A, Torres Figueroa F, Clavijo C, Arbelaez P, Cruz JC, Munoz-Camargo C. An image J plugin for the high throughput image analysis of in vitro scratch wound healing assays. PLoS One **2020**;15:e0232565
